# Conserved Distal Loop Residues in the Hsp104 and ClpB Middle Domain Contact Nucleotide-binding Domain 2 and Enable Hsp70-dependent Protein Disaggregation*

**DOI:** 10.1074/jbc.M113.520759

**Published:** 2013-11-26

**Authors:** Morgan E. DeSantis, Elizabeth A. Sweeny, David Snead, Eunice H. Leung, Michelle S. Go, Kushol Gupta, Petra Wendler, James Shorter

**Affiliations:** From the ‡ Department of Biochemistry and Biophysics and; § Biochemistry and Molecular Biophysics Graduate Group, Perelman School of Medicine, University of Pennsylvania, Philadelphia, Pennsylvania 19104 and; the ¶ Department of Biochemistry, Gene Center, Ludwig-Maximilians-Universität München, Feodor-Lynen-Strasse 25, 81377 München, Germany

**Keywords:** Molecular Chaperone, Protein Aggregation, Protein Folding, Stress, Stress Response, ClpB, DNA, Hsp104, Hsp70, Disaggreg

## Abstract

The homologous hexameric AAA^+^ proteins, Hsp104 from yeast and ClpB from bacteria, collaborate with Hsp70 to dissolve disordered protein aggregates but employ distinct mechanisms of intersubunit collaboration. How Hsp104 and ClpB coordinate polypeptide handover with Hsp70 is not understood. Here, we define conserved distal loop residues between middle domain (MD) helix 1 and 2 that are unexpectedly critical for Hsp104 and ClpB collaboration with Hsp70. Surprisingly, the Hsp104 and ClpB MD distal loop does not contact Hsp70 but makes intrasubunit contacts with nucleotide-binding domain 2 (NBD2). Thus, the MD does not invariably project out into solution as in one structural model of Hsp104 and ClpB hexamers. These intrasubunit contacts as well as those between MD helix 2 and NBD1 are different in Hsp104 and ClpB. NBD2-MD contacts dampen disaggregase activity and must separate for protein disaggregation. We demonstrate that ClpB requires DnaK more stringently than Hsp104 requires Hsp70 for protein disaggregation. Thus, we reveal key differences in how Hsp104 and ClpB coordinate polypeptide handover with Hsp70, which likely reflects differential tuning for yeast and bacterial proteostasis.

## Teaser

**Background:** How Hsp104 and ClpB coordinate polypeptide handover with Hsp70 to dissolve disordered protein aggregates is unknown.

**Results:** Conserved distal loop residues in the Hsp104 and ClpB middle domain contact NBD2 and enable Hsp70-dependent protein disaggregation.

**Conclusion:** Distal loop does not project out into solution and Hsp104 and ClpB are tuned differently for Hsp70 collaboration.

**Significance:** Understanding how protein disaggregases operate may empower strategies to counter protein-misfolding disorders.

## Introduction

**FIGURE 1.**
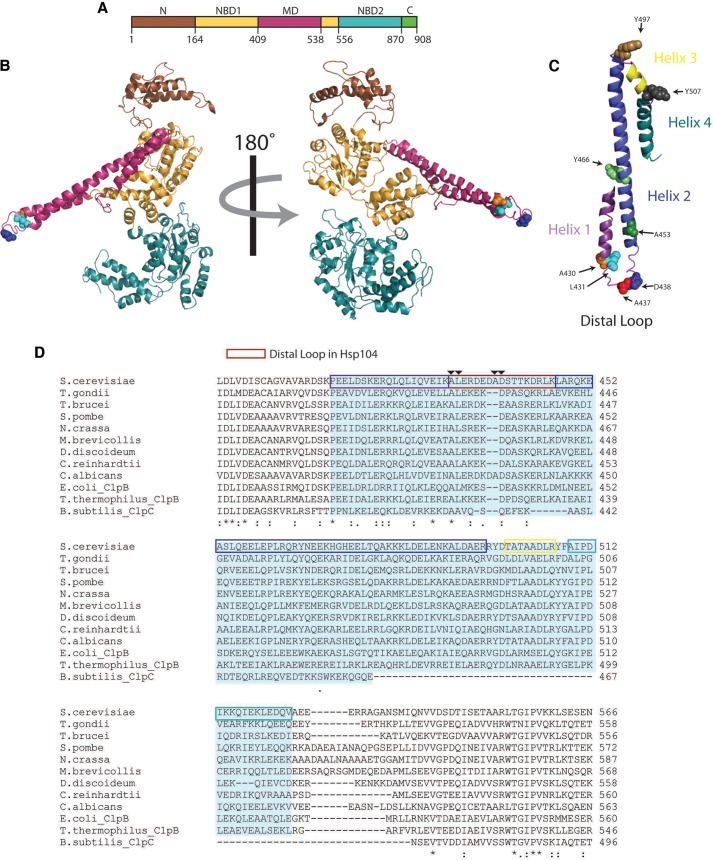
**Location of the MD distal loop.** *A,* domain schematic of Hsp104. The N-terminal domain is shown in *brown*, NBD1 in *yellow*, MD in *purple*, NBD2 in *blue*, and the C-terminal extension in *green. B,* homology model of the Hsp104 monomer with the conserved distal loop residues Ala^430^ (*orange*), Leu^431^ (*teal*), and Asp^438^ (*blue*) are shown as *spheres*. Domain coloring is as in *A. C,* homology model of the Hsp104 MD. Helix 1 is shown in *purple*, helix 2 in *blue*, helix 3 in *yellow*, and helix 4 in *green*. MD residues studied in this paper are indicated as *spheres*: Ala^430^ (*orange*), Leu^431^ (teal), Ala^437^ (*pink*), Asp^438^ (*blue*), Ala^453^ (*dark green*), Tyr^466^ (*light green*), Y497 (*dark orange*), and Tyr^507^ (*gray*). *D,* ClustalW alignment of the middle domains from several Hsp104 homologues. The MD is shaded *blue*. Helix 1 is boxed in *purple*, the distal loop region is *boxed in red*, helix 2 is *boxed in blue*, helix 3 is *boxed in yellow*, and helix 4 is *boxed in green. Arrows* indicate Ala^430^, Leu^431^, Ala^437^, and Asp^438^.

Our structural and mechanistic understanding of how the homologous hexameric AAA^+^ ATPases, Hsp104 and ClpB, disaggregate diverse substrates is limited (1, 2). Hsp104 in *Saccharomyces cerevisiae* and ClpB in *Escherichia coli* confer tolerance to thermal and chemical stress by rescuing proteins trapped in disordered aggregates (3–7). Hsp104 orthologues in other fungi, bacteria, and plants perform similar roles (8–11). Like many AAA^+^ proteins, Hsp104 and ClpB form ring-shaped hexamers with a central channel (12, 13). Each monomer contains an N-terminal domain, two AAA^+^ domains that bind and hydrolyze ATP (nucleotide-binding domain (NBD),^6^ NBD1 and NBD2)), and a coiled-coil middle domain (MD) inserted in NBD1 (Fig. 1, *A* and *B*) (1, 2, 14). Hsp104 has a C-terminal extension not found in ClpB, which is critical for Hsp104 hexamerization (Fig. 1*A*) (15).

Hsp104 and ClpB are often assumed to share a common mechanism of action due to high sequence homology in their NBDs and because some Hsp104-ClpB chimeras retain partial disaggregase activity (16–20). Hsp104 and ClpB couple energy from ATP hydrolysis to disordered aggregate dissolution, which proceeds via partial or complete translocation of substrate through the central channel of the hexamer (16, 17, 21–25). However, several dissimilarities suggest that Hsp104 and ClpB hexamers are tuned differently (2, 26). For instance, ADP or ATP binding to NBD1 is critical for ClpB hexamerization (27), whereas ADP or ATP binding to NBD2 is critical for Hsp104 to hexamerize (12, 28, 29). Furthermore, NBD1 contributes the majority of basal ATPase activity in Hsp104 (28–31), whereas both NBDs contribute in ClpB (27). ClpB and Hsp104 display differential sensitivity to activation or inhibition of disaggregase activity by the slowly hydrolysable ATP analog, ATPγS, and by mutant subunits defective in ATP hydrolysis and substrate binding (26, 32). These differences confer Hsp104 with the plasticity needed to rapidly disassemble highly stable, cross-β amyloid and prion fibrils, as well as their toxic oligomeric precursors, whereas ClpB has limited activity against these substrates (26, 33–37). These dissimilarities suggest mechanistic differences in how Hsp104 and ClpB disaggregate proteins that we are only beginning to comprehend.

Hsp104 and ClpB usually require cofactors to dissolve disordered aggregates (2). Exceptions, however, abound. Thus, ClpB from *Thermus thermophilus* and *Ehrlichia chaffeensis* disaggregate disordered aggregates without cofactors (38, 39) and Hsp104 disaggregates diverse amyloids without cofactors (26). Typically, however, to dissolve disordered aggregates Hsp104 and ClpB must collaborate with the Hsp70 chaperone system: Hsp70 (DnaK in *E. coli*) and its obligate co-chaperone Hsp40 (DnaJ in *E. coli*) (5, 6, 40), or be provided with permissive ratios of ATPγS:ATP (∼5:1–1:3 ATPγS:ATP) *in vitro* (26, 32, 41). ATPγS is thought to coordinate ATP hydrolysis in a way that enables Hsp104 and ClpB to dissolve disordered aggregates without Hsp70 (32, 41). Thus, permissive ATPγS:ATP ratios allow the disaggregation of disordered aggregates by Hsp104 or ClpB to be studied in the absence of Hsp70 (32, 41). This, in turn, allows mutations to be defined in Hsp104 or ClpB that specifically ablate Hsp70 collaboration but do not affect the intrinsic disaggregase activity of Hsp104 or ClpB. By intrinsic disaggregase activity we mean disaggregase activity observed in the absence of Hsp70 but in the presence of ATPγS:ATP or in the presence of ATP alone.

Precisely how Hsp70 and Hsp104 coordinate substrate handling in protein disaggregation is unclear (42). Hsp104 cannot collaborate with bacterial DnaK and ClpB cannot collaborate with eukaryotic Hsp70 (2, 5, 18–20, 43). Hsp70 and DnaJ collaborate with Hsp104, whereas DnaK and Hsp40 cannot (5). Thus, Hsp70 determines specificity and might interact with Hsp104 directly (44–46).

The coiled-coil MD of Hsp104 and ClpB plays a key role in Hsp70 collaboration (18–20) and is comprised of four helices (Fig. 1, *B* and *C*). Artificial Hsp104 chimeras containing the ClpB MD (in place of the Hsp104 MD) weakly collaborate with DnaK to disaggregate disordered aggregates, whereas artificial ClpB chimeras containing the Hsp104 MD can interface with eukaryotic Hsp70 (18–20). Transplanting artificial chimeric MDs into ClpB suggested that helices 2 and 3 of the ClpB MD are critical for DnaK collaboration, whereas helix 4 is not and helix 1 plays an important but not critical role (2, 19). By contrast, helices 1, 2, and 3 of the Hsp104 MD are important for collaboration with eukaryotic Hsp70 (2, 19). Portions of helix 2 and 3 (amino acids 481 to 500) of the ClpB MD might transiently engage Hsp70 directly (44, 45). Moreover, specific mutations in helix 2 and 3 of ClpB (*e.g.* Y503A) abolished ClpB collaboration with DnaK (44, 47, 48). A more N-terminal stretch of MD helix 2 of Hsp104 (amino acids 477–488) transiently engages Hsp70 and replacing three lysines (Lys^480^, Lys^481^, and Lys^482^) in this region with glutamate or alanine ablates Hsp104 disaggregase activity in the presence of Hsp70 (46).

Here, we build on previous studies and define the function and location of the distal loop region (residues 430–446) between helix 1 and 2 of the MD of Hsp104 (Fig. 1, *B–D*). The function of the MD distal loop has been largely ignored and its location in the hexamer remains controversial due to radically different placements in conflicting hexameric models of Hsp104 and ClpB (14, 48–50). Indeed, there is no high resolution structure of Hsp104 or ClpB hexamers, and two conflicting models have been proposed based on cryo-electron microscopy (EM) studies (2, 14, 49–52). Here, we uncover several Hsp104 variants with mutations in the MD distal loop that retain WT intrinsic disaggregase activity (*i.e.* are activated by ATPγS:ATP and inactive with ATP alone without Hsp70) but are unable to collaborate with Hsp70. This distal loop function is conserved and enables ClpB collaboration with DnaK. We show that the distal loop does not stably contact Hsp70, but regulates Hsp104 and ClpB activity via an autoinhibitory intramolecular contact with NBD2. This NBD2-MD contact is inconsistent with structural models of ClpB and Hsp104 hexamers in which the distal loop projects out into solution away from the hexamer (14, 46, 49, 53) or invariably contacts NBD1 of the neighboring subunit (48). We establish that ClpB requires DnaK more stringently than Hsp104 requires Hsp70 to dissolve disordered aggregates. Thus, we reveal a crucial unanticipated role for the MD distal loop of Hsp104 and ClpB in Hsp70 collaboration, as well as unexpected differences in how these protein disaggregases dissolve disordered aggregates.

## EXPERIMENTAL PROCEDURES

### 

#### 

##### Protein Expression and Purification

ClpB and Hsp104 mutants were generated via QuikChange Lightning mutagenesis (Agilent). ClpB variants were expressed as C terminally His_6_-tagged constructs from a pDS56/RBSII plasmid in M15 cells (Qiagen). Hsp104 variants, including isolated MD (amino acids 407–527), were expressed and purified as N terminally His_6_-tagged constructs (54) or as untagged constructs. Briefly, Hsp104 in pNOTAG (31) was transformed into BL21-DE3 RIL cells (Agilent). Expression was induced at an *A*_600_ of 0.4–0.6 with 1 mm isopropyl 1-thio-β-d-galactopyranoside for 15–18 h at 15 °C. Cells were harvested via centrifugation (4,000 × *g*, 4 °C, 20 min), resuspended in lysis buffer (50 mm Tris-HCl, pH 8, 10 mm MgCl_2_, 2.5% glycerol (w/v), 2 mm β-mercaptoethanol, 5 μm pepstatin, and 1 Mini-EDTA free protease tablets per 50 ml (Roche Applied Science)), and lysed via treatment with 20 mg of hen egg lysozyme per 1 liter of cells followed by probe sonication. Cell debris was removed via centrifugation at 16,000 × *g* at 4 °C. The supernatant was applied to 3 ml of Affi-Gel Blue (Bio-Rad) per 1 liter of cell culture. Supernatant and resin were rotated at 20 rpm at 4 °C for 3 h. Resin was then washed 4 times with wash buffer (50 mm Tris-HCl, pH 8, 10 mm MgCl_2_, 100 mm KCl, 2.5% glycerol (w/v), 2 mm β-mercaptoethanol). Hsp104 was eluted with high-salt buffer (wash buffer with 1 m KCl). Hsp104 was then purified further by Resource Q anion-exchange chromatography and size-exclusion chromatography (54). The isolated MD (amino acids 407–527) was cloned into a derivative of pET DUET (55) to yield a N terminally GST-tagged MD (GST-MD). This construct was transformed into BL21-DE3 RIL cells, which were grown until mid-log phase and then induced with 1 mm isopropyl 1-thio-β-d-galactopyranoside for 2.5 h at 37 °C. GST-MD was purified using glutathione-Sepharose according to the manufacturer's instructions (GE Healthcare). Firefly luciferase was from Sigma. Hsc70, Hdj2, DnaK, DnaJ, and GrpE were from Enzo Life Sciences. Unless otherwise stated, ClpB and Hsp104 concentrations refer to the hexamer.

##### Luciferase Reactivation

Luciferase reactivation was performed as described (5). To assemble aggregates, firefly luciferase (50 μm) in luciferase refolding buffer (25 mm HEPES-KOH, pH 7.4, 150 mm KAOc, 10 mm MgAOc, 10 mm DTT) with 6 m urea was incubated at 30 °C for 30 min. The sample was then rapidly diluted 100-fold into luciferase refolding buffer, divided into 100-μl aliquots, snap frozen in liquid N_2_, and stored at −80 °C. For reactivation assays, aggregated luciferase (50 nm) was incubated with Hsp104 (1 μm), Hsc70 (1 μm), and Hdj2 (1 μm) plus ATP (5.1 mm) and an ATP regeneration system (1 mm creatine phosphate, 0.25 μm creatine kinase (Roche Applied Science)) for 90 min at 25 °C. In some experiments, Hsc70 and Hdj2 were replaced with 4 mm ATPγS and 1.1 mm ATP. In other experiments, isolated His_6_-MD (6 or 36 μm) was added. For luciferase reactivation by ClpB, Hsp104, Hsc70, and Hdj2 were replaced with ClpB (0.167 μm), DnaK (0.167 μm), DnaJ (0.033 μm), and GrpE (0.0167 μm) and the reaction was incubated for 60 min at 25 °C. In some experiments, DnaK, DnaJ, and GrpE were replaced by 2.5 mm ATPγS and 2.6 mm ATP. For experiments with disulfide cross-linked Hsp104 or ClpB variants, luciferase aggregates were assembled and reactivated as above except that DTT was omitted from all buffers. Luciferase activity was assessed with a luciferase assay system (Promega). Luminescence was monitored on a Tecan Safire^2^ or Infinite M1000 plate reader. For doping experiments, the conditions for ClpB and Hsp104 were kept as similar as possible. Thus, GrpE was present (0.167 μm) or absent from the reaction and 1 μm ClpB, 1 μm DnaK, and 1 μm DnaJ were used. Hsp104, Hsc70, and Hdj2 were used as described above. Total Hsp104 or ClpB was comprised of WT or mutant or a 1:5, 2:4, 3:3, 4:2, and 5:1 mixture of the two. To ensure statistical subunit mixing, WT and mutant Hsp104 or ClpB mixtures were equilibrated for 15 min on ice prior to addition to the reaction (26).

##### In Vivo Thermotolerance Assay

W303 Δ*hsp104* (*MATa*, *can1–100*, *his3–11,15*, *leu2–3,112*, *trp1–1*, *ura3–1*, *ade2–1*, *hsp104*:*kanMX4*) yeast were transformed with a centromeric plasmid bearing the HSP104 promoter, pHSE, or pHSE encoding Hsp104 or the indicated Hsp104 variant (4). Strains were grown in SD-ura media to an *A*_600_ of 0.55 and then incubated at 37 °C for 30 min to induce Hsp104 variant expression. Cells were then heat shocked at 50 °C for 0–20 min. After heat shock, cells were transferred to ice for 2 min and spotted on SD-ura in a 5-fold dilution series. Plates were incubated at 30 °C for 2 days. Alternatively, cells were plated onto SD-ura and allowed to recover for 3 days at 30 °C to assess viability. Colonies were counted using an aCOLyte automated colony counter (Synbiosis). Immunoblot showed that each Hsp104 protein was expressed at similar levels.

##### Circular Dichroism (CD)

Hsp104 variants were dialyzed into CD buffer (50 mm Na_3_PO_4_, 50 mm K_3_PO_4_, and 10 mm MgAOc, pH 7) and the concentration was adjusted to 1 μm monomer. Data were collected on an Aviv Biomedical Inc. CD spectrophotometer (model 410). Samples were scanned from 260 to 190 nm with a 1-nm step size and a 15-s averaging time at 25 °C.

##### Size Exclusion Chromatography

Hsp104 variants were exchanged into running buffer (40 mm HEPES-KOH, pH 7.4, 150 mm KCl, 10 mm MgCl_2_, and 1 mm DTT). The Hsp104 concentration was adjusted to 35 μm monomer and 100 μl of sample was fractionated by a Superose 6 10/300 column (GE Healthcare).

##### ATPase Assay

Hsp104 or ClpB (0.25 μm monomer) were incubated for 12 min at 25 °C (for Hsp104) or 30 min at 25 °C (for ClpB) with ATP (1 mm). ATPase activity was determined by the amount of inorganic phosphate released using a malachite green phosphate detection kit (Innova).

##### Hsp70 Pull-down with GST-MD

GST or GST-MD was coupled to glutathione-Sepharose at 2.5 μg/μl and incubated for 15 min on ice with Hsc70 (3.5 μm) in storage buffer (40 mm HEPES, pH 7.4, 150 mm KCl, 20 mm MgCl_2_, 10% glycerol (w/v), 1 mm DTT). Recovered beads were washed 4 times in storage buffer, with the second wash containing 1 mm ATP, and then eluted with storage buffer plus 20 mm reduced glutathione. Samples were processed for SDS-PAGE and Coomassie staining.

##### Tryptophan Fluorescence

Hsp104 variants were dialyzed into low salt buffer (20 mm HEPES-KOH, pH 7.4, 20 mm NaCl, 10 mm MgCl_2_, 1 mm DTT) to ensure that all proteins were hexameric, even in the absence of nucleotide (30). The concentration was adjusted to 5.5 μm monomer and samples were left in the apo state or incubated with 5 mm ATP, ADP, ATPγS, or AMP-PNP (a non-hydrolysable ATP analog). Samples were excited at 295 nm (4-mm bandwidth) and emission was collected from 305 to 505 nm (3-nm bandwidth) with a 1-nm step size using a Fluorolog-3–21 Jobin-Yvon Spex Instrument SA (Edison, NJ). Spectra of buffer matched controls were subtracted.

##### X-ray Footprinting

WT Hsp104 was purified as above except the final step of purification was gel filtration using a Superdex 200 column (GE Healthcare) equilibrated in 50 mm sodium cacodylate, pH 7.0, 140 mm KCl, and 10 mm MgCl_2_. Hsp104 was diluted immediately prior to use to 10 μm in 50 mm sodium cacodylate, pH 7.0, 140 mm KCl, 10 mm MgCl_2_, supplemented with 1 mm ADP or ATPγS (for the hexamer). Oxidation of Alexa 488 (Invitrogen) was used to determine optimal exposure time for each buffer and provide normalization constants. Samples were exposed to a mirror-focused synchrotron x-ray beam (5.5 megarad angle, focus value of 6 mm) at the X28C beamline of the National Synchrotron Light Source at Brookhaven National Laboratory for 0–20 ms. Exposure time was controlled by flow rate through the flow cell of a KinTek (Austin, TX) stopped flow apparatus (56). Oxidation was immediately quenched with methioninamide (10 mm) and samples were snap frozen in liquid N_2_. Irradiated protein samples were thawed on ice, diluted to 1 μm in 5 mm HEPES-NaOH, pH 7.0, 140 mm KCl, 10 mm MgCl_2_, 1 mm DTT, 1% TFA, and injected into an on-line fragmentation-separation/MS analysis system. Samples were passed through an immobilized pepsin column onto a C18 trap column. Following a 3-min wash, peptides were eluted with a non-linear elution gradient optimized for the highly charged Hsp104 peptide fragments (6 μl/min non-linear 2–50% acetonitrile gradient with 0.1% formic acid at pH 2 and 0 °C) and passed through an analytical C18 HPLC column before injection by electron spray ionization (ESI) to an LTQ Orbitrap XL mass spectrometer (Thermo Scientific). To enhance peptide identification, selected peptide ions (the four most abundant in each scan) were fragmented by CID and measured in the LTQ stage. A “peptide pool” of unmodified peptides was obtained by combining high probability peptides identified from 0-ms MS runs. A program used for the identification of H/D exchange data, ExMS (57), was modified to identify oxidatively modified peptides. A list of theoretically modified peptides was created using the unmodified peptide pool and the list of potential modifications for each amino acid (58), and the MS spectra was searched for the theoretical masses. Results were confirmed by searching the spectra with a modified ExMS (57) analysis against the Hsp104 primary sequence. The fraction of unmodified peptide was calculated by taking the intensity of the unmodified peak over the sum of the unmodified peptide and all singly modified versions of the peptide. Data were fit to a first order exponential to determine the rate of modification, which was then normalized using the Alexa 488 decay data.

##### Disulfide and 1,4-Bis-maleimidobutane (BMB) Cross-linking

For cross-linking, we employed an Hsp104 variant, Hsp104^6CS^, where all six of the native cysteines of Hsp104 (positions: 209, 400, 643, 718, 721, and 876) are mutated to serine. Engineered cysteines were introduced into this background. For ClpB, native cysteines were not mutated to serine. After purification, samples were exchanged into running buffer without DTT. Protein concentrations were adjusted to 60 μm and samples were incubated with 1 mm diamide for 30 min at room temperature. Samples were exchanged into running buffer without DTT to remove excess diamide. Disulfide cross-link formation was assessed via SDS-PAGE on 4–20% Tris-HCl non-reducing gel in comparison to non-cross-linked samples (Bio-Rad). Gels were Coomassie stained and intramolecular disulfide formation caused proteins to migrate more slowly (47). Quantification of band intensities was determined by densitometry. In some cases, Ellman's reagent (Thermo Scientific Pierce) was used to quantify the amount of free sulfhydryls and thus disulfide formation according to the manufacturer's instructions. In other experiments, the flexible 11-Å cross-linker BMB was used to make a covalent intramolecular cross-link between A430C to F630C in Hsp104^6CS/A430C/F630C^ according to the manufacturer's instructions (Thermo Scientific Pierce). For all activity assays with disulfide cross-linked proteins, DTT was omitted unless specifically stated.

##### Small Angle and Wide Angle X-ray Scattering (SAXS and WAXS)

SAXS and WAXS data were collected simultaneously at beamline X9 at the National Synchrotron Light Source (NSLS, Upton, NY) at 10 °C by two overlapping detectors, a Mar 165 CCD SAXS detector 3.4 m from the sample, and a custom built Photonic Science CCD WAXS detector. The two-dimensional scattering images collected on the CCD detectors were circularly averaged using software developed at the beamline to yield one-dimensional scattering profiles as a function of momentum transfer *q* (*q* = 4πsin(θ)/λ, where 2π is the scattering angle and λ is the wavelength). The x-ray wavelength was 0.855 Å and the angular range collected was 0.00550 ≤ *q* ≥ 1.0060. The sample cell contained a glass capillary sealed across the evacuated chamber. Protein samples and matching buffer solutions were flowed through the capillary during exposure to reduce radiation damage. For data collection, 30 μl of the protein sample at concentrations between 1.5 and 6 mg/ml or matching buffer (20 mm HEPES-KOH, pH 7.4, 140 mm KCl, 10 mm MgCl_2_, 2 mm DTT, 2 mm ATP) was exposed for 180 s, subdivided into three 60-s exposures of 10 μl. After each measurement the capillary was washed thoroughly and purged with compressed nitrogen.

The raw scattering data were scaled and buffer was subtracted using the program PRIMUS (59). Each individual scattering curve was visually inspected for radiation damage and aggregation prior to averaging, including Guinier and Kratky plot analysis. Averaged scattering curves from the SAXS and WAXS detectors were scaled and merged in PRIMUS to yield a low-noise composite curve. The radii of gyration (*R_g_*) were initially calculated using Guinier plots (60). *P*(*r*) distance distribution functions were calculated by the program GNOM (61) using an indirect Fourier transform. The maximum dimension of the particle (*D*_max_) was determined by examining the quality of fit to the experimental data for a *D*_max_ range of 180 to 280 Å, varied in 5-Å increments. Fits were performed to maximize the total estimate figure, to minimize the discrepancy between calculated and experimental profiles, and to optimize the visual properties of the shape distribution function. Values for *R_g_* were computed from the second moment of the *P*(*r*) and compared favorably to those calculated using Guinier plots.

##### Mathematical Modeling of Heterohexamer Ensemble Activity

We simulated the distribution of WT and mutant subunits within a given population of Hsp104 or ClpB hexamers as described (26). Briefly, we employed the binomial distribution,

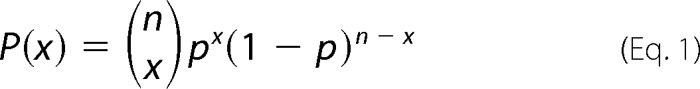
 where *P* is the probability that a hexamer (thus, *n* = 6) contains *x* mutant subunits and *p* is the probability that a mutant subunit is incorporated. Experiments demonstrated that mutant and WT subunits have a similar probability of being incorporated into a hexamer (26). Consequently, *p* is calculated as the molar ratio of mutant and WT protein present.

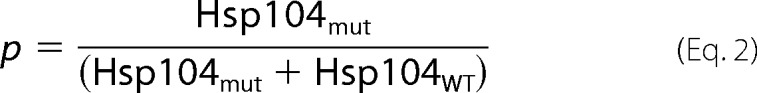
 Therefore, for any specified percentage of mutant subunits the probability distribution of Hsp104 hexamers containing 0, 1, 2, 3, 4, 5, or 6 mutant subunits can be derived (Fig. 9*A*). Activity *versus p* plots (Fig. 9*B*) could then be generated assuming each WT subunit makes an equal contribution to the total activity (one-sixth per subunit). Consequently, if subunits within the hexamer operate independently then activity should decline in a linear manner upon incorporation of defective mutant subunits. Conversely, if subunits are coupled then a specific number of subunits will be sufficient to eliminate activity. Thus, zero activity is assigned to hexamers that are in breach of a specific threshold number of mutant subunits. In this way, we can generate activity *versus p* plots if we assume that 1 or more, 2 or more, 3 or more, 4 or more, or 5 or more mutant subunits are required to eliminate activity (26, 38).

## RESULTS

### 

#### 

##### The Y507A Mutation in Hsp104 Has Pleiotropic Effects on Disaggregase Functionality

**FIGURE 2.**
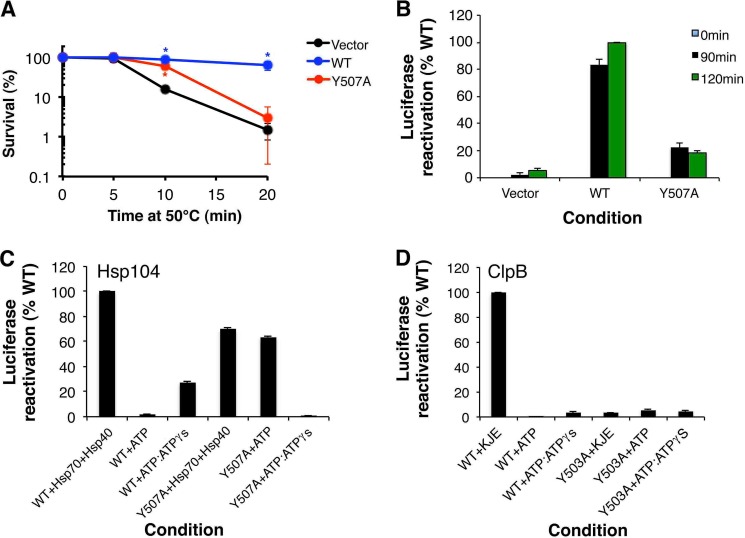
**Hsp104^Y507A^ is a hypomorph with altered intrinsic disaggregase activity.** *A*, Δ*hsp104* yeast cells harboring empty pHSE vector (*black markers*), pHSE-Hsp104 (*blue markers*), or pHSE-Hsp104^Y507A^ (*red markers*) were grown to mid-log phase in SD-ura liquid. Prior to the 50 °C heat treatment, matched cultures were preincubated at 37 °C for 30 min. Following treatment at 50 °C for 0–20 min cells were transferred to ice, diluted in ice-cold SD-ura, immediately plated on SD-ura, and allowed to recover for 3 days at 30 °C and cell viability was assessed. Values represent mean ± S.E. (*n* = 4). A one-way ANOVA with the post hoc Dunnett's multiple comparisons test was used to compare vector alone to the Hsp104^WT^ and Hsp104^Y507A^ constructs (* denotes *p* < 0.05). *B,* Δ*hsp104* yeast cells harboring pGPD-LuxAB (encoding a temperature-sensitive luciferase fusion protein) and either empty vector (*pHSE*), pHSE-Hsp104, or pHSE-Hsp104^Y507A^ were grown to mid-log phase in SD-his-ura liquid. Matched cultures were preincubated at 37 °C for 30 min and then incubated at 44 °C for 50 min. Cycloheximide (10 μg/ml) was then added and cultures were incubated for a further 10 min at 44 °C. Cells were then shifted to 30 °C and luciferase activity was measured at 0, 90, and 120 min. Luciferase activity was expressed as the % of WT Hsp104 condition after 120 min. Values represent mean ± S.E. (*n* = 3). *C,* urea-denatured firefly luciferase aggregates were incubated for 90 min at 25 °C with Hsp104 (1 μm) or Hsp104^Y507A^ (1 μm) in the presence of ATP (5.1 mm), a mixture of ATP (1 mm) and ATPγS (4 mm), or Hsc70 (an Hsp70) (1 μm) and Hdj2 (an Hsp40) (1 μm) plus ATP (5.1 mm). Reactivation of luciferase was then determined by luminescence and converted to % WT disassembly activity (activity of 1 μm WT Hsp104 in the presence of Hsc70 and Hdj2). Values represent mean ± S.E. (*n* = 3). *D,* urea-denatured firefly luciferase aggregates were incubated for 60 min at 25 °C with ClpB (0.167 μm) or ClpB^Y503A^ (0.167 μm) in the presence of ATP (5.1 mm), a mixture of ATP (2.5 mm) and ATPγS (2.5 mm), or DnaK (1 μm), DnaJ (0.033 μm), and GrpE (0.0167 μm) plus ATP (5.1 mm). Reactivation of luciferase was then determined by luminescence and converted to % WT ClpB activity (activity of 0.167 μm WT ClpB in the presence of DnaK, DnaJ, and GrpE). Values represent mean ± S.E. (*n* = 3).

First, we assessed the MD helix 3 variant, Hsp104^Y507A^ (Fig. 1*C*) in induced thermotolerance in yeast (4). Hsp104^Y507A^ is the equivalent of ClpB^Y503A^, which is specifically defective in DnaK collaboration (44, 47). In preconditioned Δ*hsp104* cells, Hsp104^Y507A^ provided limited thermotolerance (Fig. 2*A*). After 20 min at 50 °C, Hsp104^Y507A^ conferred survival levels similar to the vector control (Fig. 2*A*). However, Hsp104^Y507A^ was not completely inactive and after 10 min at 50 °C provided significant thermotolerance similar to WT Hsp104 (Fig. 2*A*). WT Hsp104 and Hsp104^Y507A^ were expressed at similar levels (data not shown). Hsp104^Y507A^ reactivated aggregated luciferase *in vivo* although not as effectively as WT Hsp104 (Fig. 2*B*). Thus, contrary to expectations from data obtained with ClpB^Y503A^ (44, 47), Hsp104^Y507A^ is hypomorphic and not a null variant.

Can Hsp104^Y507A^ collaborate with Hsp70? To answer this question, we assessed the ability of Hsp104^Y507A^ to reactivate urea-denatured luciferase aggregates *in vitro* (5). In the presence of Hsp70 and Hsp40, Hsp104^Y507A^ was ∼70% as active as WT Hsp104 under the same conditions (Fig. 2*C*). This finding explains why Hsp104^Y507A^ is partially active *in vivo* (Fig. 2, *A* and *B*). However, Hsp104^Y507A^ was also highly active in the presence of ATP in the absence of Hsp70 and Hsp40, whereas WT Hsp104 was inactive (Fig. 2*C*). In the presence of ATP, Hsp70 and Hsp40 only modestly stimulated (∼10%) Hsp104^Y507A^ disaggregase activity (Fig. 2*C*). Moreover, substituting a permissive ratio of ATPγS:ATP in place of Hsp70 and Hsp40 inhibited Hsp104^Y507A^, whereas WT Hsp104 was activated (Fig. 2*C*). Thus, the Y507A mutation is pleiotropic and causes alterations in the intrinsic disaggregase activity of Hsp104 as well as reduced ability to collaborate with Hsp70.

By contrast, ClpB^Y503A^ is selectively impaired in the ability to collaborate with DnaK, DnaJ, and GrpE (KJE). ClpB^Y503A^ has minimal activity in the presence of ATP and absence of DnaK, DnaJ, and GrpE (44, 47), and is activated to WT levels with a permissive ratio of ATP:ATPγS (Fig. 2*D*). Note that the relative activation of ClpB by ATP:ATPγS is low compared with ClpB activity in the presence of DnaK, DnaJ, and GrpE (Fig. 2*D*), whereas in Hsp104 it is considerably higher (Fig. 2*C*). Thus, for optimal activity ClpB requires Hsp70 more stringently than Hsp104. These findings indicate that mutation of the conserved tyrosine in helix 3 of the MD (Tyr^503^ in ClpB, Tyr^507^ in Hsp104) to alanine has different functional consequences for Hsp104 *versus* ClpB.

##### Specific Mutations in the Distal Loop of Hsp104 Selectively Ablate Hsp70 Collaboration

We sought non-pleiotropic mutations in Hsp104 that specifically ablated collaboration with Hsp70, whereas having no effect on the intrinsic disaggregase activity of Hsp104 against disordered aggregates. Such Hsp104 variants would be unable to disaggregate disordered aggregates in the presence of Hsp70 and Hsp40. However, they would still disaggregate disordered aggregates in the presence of ATPγS:ATP alone and be inactive with ATP alone without Hsp70. We hypothesized that the distal loop (residues 430–446 in Hsp104) between helix 1 and 2 of the MD (Fig. 1*C*) might be important for Hsp70 collaboration because in another Hsp100, ClpC, this region contacts co-factors (62). Moreover, in peptide arrays, Hsp70 interacts with a peptide (residues 435–448) in this region (46). Thus, we mutated various residues in the distal loop and assessed functionality (Fig. 1, *C* and *D*).

We focused on three distal loop residues: Ala^430^, Leu^431^, and Asp^438^, because they are highly conserved (Fig. 1, *B–D*). Initially, we made a drastic change and introduced the bulky, hydrophobic residue, tryptophan, in an effort to perturb function. Importantly, CD revealed that Hsp104^A430W^, Hsp104^L431W^, and Hsp104^D438W^ were very similar to WT Hsp104 in terms of secondary structure (data not shown). Moreover, the isolated MD bearing the A430W mutation had similar secondary structure to WT MD (data not shown). Hsp104^A430W^, Hsp104^L431W^, and Hsp104^D438W^ assembled into hexamers just like WT Hsp104 (data not shown). Thus, these mutations do not grossly perturb the Hsp104 structure.

**FIGURE 3.**
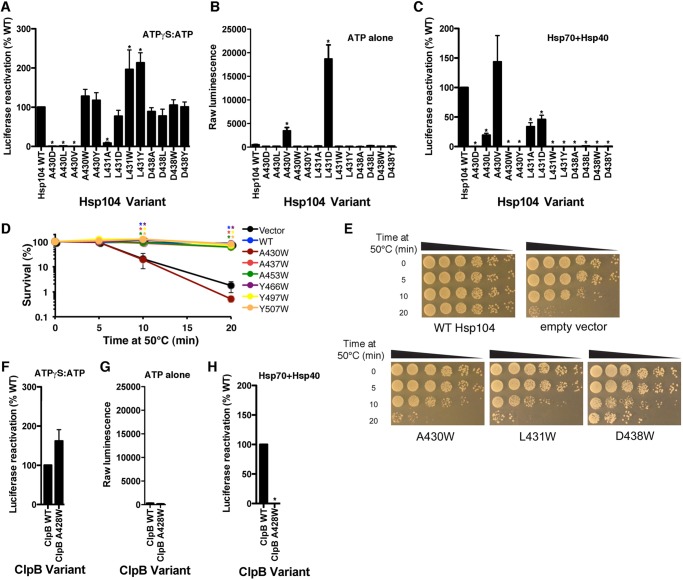
**Hsp104^A430W^, Hsp104^L431W^, and Hsp104^D438W^ are specifically defective in Hsp70 collaboration.** *A,* urea-denatured firefly luciferase aggregates were incubated for 90 min at 25 °C with Hsp104 (1 μm) or the indicated Hsp104 variant (1 μm) plus ATP (1 mm) and ATPγS (4 mm). Luciferase reactivation was then determined and converted to % WT disaggregase activity in the presence of ATPγS:ATP. Values represent mean ± S.E. (*n* = 3–10). A one-way analysis of variance with the post hoc Dunnett's multiple comparisons test was used to compare Hsp104^WT^ to Hsp104 variants (* denotes *p* < 0.05). *B,* reactions were performed as in *A* except that the mixture of ATP (1 mm) and ATPγS (4 mm) was replaced with ATP (5.1 mm). Values represent mean ± S.E. (*n* = 3–13). A one-way analysis of variance with the post hoc Dunnett's multiple comparisons test was used to compare Hsp104^WT^ to Hsp104 variants (* denotes *p* < 0.05). *C,* reactions were performed as in *A,* except that ATP (1 mm) and ATPγS (4 mm) were replaced with Hsc70 (1 μm) and Hdj2 (1 μm) plus ATP (5.1 mm). Luciferase reactivation was then determined and converted to % WT disaggregase activity in the presence of the Hsc70 and Hdj2. Values represent mean ± S.E. (*n* = 3–10). A one-way analysis of variance with the post hoc Dunnett's multiple comparisons test was used to compare Hsp104^WT^ to Hsp104 variants (* denotes *p* < 0.05). *D,* Δ*hsp104* yeast cells harboring empty pHSE vector (*black markers*) or the indicated pHSE-Hsp104 variant (WT, A430W, A437W, A453W, Y466W, Y497W, or Y507W) were grown to mid-log phase in SD-ura liquid. Prior to the 50 °C heat treatment, matched cultures were preincubated at 37 °C for 30 min. Following treatment at 50 °C for 0–20 min cells were transferred to ice, diluted in ice-cold SD-ura, immediately plated on SD-ura, and allowed to recover for 3 days at 30 °C and cell viability was assessed. Note that all the Hsp104 variants retain WT levels of activity except Hsp104^A430W^, which behaves as a null allele. Values represent mean ± S.E. (*n* = 3). We employed a one-way analysis of variance with the post hoc Dunnett's multiple comparisons test was used to compare vector alone to each Hsp104 variant (* denotes *p* < 0.05). *E,* Δ*hsp104* yeast cells harboring empty pHSE vector (*black markers*) or the indicated pHSE-Hsp104 variant (WT, A430W, L431W, or D438W) were grown to mid-log phase in SD-ura liquid. Prior to the 50 °C heat treatment, matched cultures were preincubated at 37 °C for 30 min. Following treatment at 50 °C for 0–20 min cells were transferred to ice, and spotted in 5-fold serial dilutions on SD-ura. Cells were allowed to recover for 3 days at 30 °C and cell viability was assessed. *F,* urea-denatured firefly luciferase aggregates were incubated for 60 min at 25 °C with ClpB (0.167 μm) or ClpB^A428W^ plus ATP (2.5 mm) and ATPγS (2.5 mm). Luciferase reactivation was then determined and converted to % WT disaggregase activity in the presence of ATPγS:ATP. Values represent mean ± S.E. (*n* = 4). A *t* test was used to compare ClpB to ClpB^A428W^ (* denotes *p* < 0.05). *G,* reactions were performed as in *F* except that the mixture of ATP and ATPγS was replaced with ATP (5.1 mm). Values represent mean ± S.E. (*n* = 3). A *t* test was used to compare ClpB to ClpB^A428W^ (* denotes *p* < 0.05). *H,* reactions were performed as in *F* except that the mixture of ATP and ATPγS was replaced with DnaK (1 μm), DnaJ (0.033 μm), and GrpE (0.0167 μm) plus ATP (5.1 mm). Reactivation of luciferase was then determined by luminescence and converted to % WT disaggregase activity in the presence of the Hsp70 chaperone system. Values represent mean ± S.E. (*n* = 4). A *t* test was used to compare ClpB to ClpB^A428W^ (* denotes *p* < 0.05).

Hsp104^A430W^ and Hsp104^D438W^ retained WT levels of intrinsic disaggregase activity in the presence of a permissive ATPγS:ATP ratio (Fig. 3*A*). Intriguingly, Hsp104^L431W^ displayed elevated intrinsic disaggregase activity with ATPγS:ATP (Fig. 3*A*). Moreover, like WT Hsp104, these Hsp104 variants possessed no disaggregase activity in the presence of ATP and absence of Hsp70 and Hsp40 (Fig. 3*B*). Thus, in contrast to Hsp104^Y507A^, in the absence of Hsp70 the distal loop variants Hsp104^A430W^, Hsp104^A430Y^, and Hsp104^D438W^ are activated by ATPγS:ATP and inactive with ATP alone similar to WT Hsp104.

In striking contrast to WT Hsp104, Hsp104^A430W^, Hsp104^L431W^, and Hsp104^D438W^ were unable to collaborate with Hsp70 and Hsp40 to reactivate aggregated luciferase (Fig. 3*C*). Indeed, in the presence of Hsp70 and Hsp40 plus ATP, Hsp104^A430W^, Hsp104^L431W^, and Hsp104^D438W^ were completely inactive (Fig. 3*C*). We obtained similar results when we substituted these positions with tyrosine instead of tryptophan (Fig. 3, *A–C*). Hsp104^A430Y^ and Hsp104^D438Y^ retained WT levels of intrinsic disaggregase activity in the presence of ATPγS:ATP, whereas Hsp104^A431Y^ disaggregase activity with ATPγS:ATP was elevated (Fig. 3*A*). Moreover, Hsp104^A430Y^, Hsp104^A431Y^, and Hsp104^D438Y^ were completely inactive in the presence of ATP alone (Fig. 3*B*), or when combined with Hsp70 and Hsp40 (Fig. 3*C*). These are the first Hsp104 mutants that are: (*a*) unable to collaborate with Hsp70 and Hsp40; (*b*) inactive with ATP alone; and (*c*) retain disaggregase activity elicited by ATPγS:ATP. Thus, conserved MD distal loop residues are critical for Hsp70 collaboration.

##### Conserved Distal Loop Residues Regulate Hsp104 Disaggregase Activity

Next, we explored the effect of different missense mutations at position Ala^430^ (Fig. 1, *C* and *D*) on intrinsic disaggregase activity and ability to collaborate with Hsp70 and Hsp40. Interestingly, Hsp104^A430D^ and Hsp104^A430L^ displayed reduced disaggregase activity elicited by ATPγS:ATP (Fig. 3*A*) and reduced Hsp70-dependent disaggregation (Fig. 3*C*). By contrast, Hsp104^A430V^ was active in the presence of Hsp70 and Hsp40 (Fig. 3*C*), and was also slightly active in the presence of ATP alone (∼2.3% of WT-Hsp104 with Hsp70 and Hsp40; Fig. 3*B*). However, Hsp104^A430V^ had lost intrinsic disaggregase activity in the presence of ATPγS:ATP (Fig. 3*A*). Thus, depending upon the precise substitution, mutation of Ala^430^ can inhibit Hsp70 collaboration (*e.g.* Trp), inhibit intrinsic disaggregase activity in the presence of ATPγS:ATP (*e.g.* Val), or both (*e.g.* Asp or Leu). These findings suggest that Ala^430^ plays a key role in regulating Hsp104 disaggregase activity.

We next explored different missense mutations at Leu^431^ (Fig. 1*C*), which is highly conserved (Fig. 1*D*). Against disordered luciferase aggregates, Hsp104^L431A^ displayed lowered activity in Hsp70-dependent disaggregation (Fig. 3*C*) and Hsp70-independent disaggregation elicited by ATPγS:ATP (Fig. 3*A*). The reduction was more severe for intrinsic disaggregase activity elicited by ATPγS:ATP (Fig. 3, *A* and *C*). By contrast, Hsp104^L431D^ displayed mildly reduced Hsp70-dependent disaggregase activity (Fig. 3*C*), whereas the intrinsic disaggregase activity in the presence of ATPγS:ATP was not significantly affected (Fig. 3*A*). Interestingly, Hsp104^L431D^ exhibited some ability to reactivate aggregated luciferase with ATP alone in the absence of Hsp70 and Hsp104 (∼12.2% of WT-Hsp104 with Hsp70 and Hsp40), whereas WT Hsp104 is completely inactive under these conditions (Fig. 3*B*). Thus, Leu^431^ also regulates Hsp104 disaggregase activity.

We also explored Asp^438^ variants (Fig. 1*C*). Mutation of Asp^438^ to Trp, Tyr, Leu, or Ala had the same effect: Hsp70-dependent reactivation of aggregated luciferase was ablated, whereas intrinsic disaggregase activity in the presence of ATPγS:ATP or ATP alone was unaltered (Fig. 3, *A–C*). Thus, Asp^438^ plays a critical role in Hsp70 collaboration, but can be substituted and not affect the ability of Hsp104 to process aggregated substrates *per se*. These studies suggest that Ala^430^, Leu^431^, and Asp^438^ all make important contributions to Hsp70 collaboration. Moreover, Ala^430^ and Leu^431^ tightly regulate Hsp104 disaggregase activity.

##### Hsp104^A430W^, Hsp104^L431W^, and Hsp104^D438W^ Are Defective in Induced Thermotolerance in Vivo

We focused on the tryptophan variants, Hsp104^A430W^, Hsp104^L431W^, and Hsp104^D438W^, because they are specifically defective in Hsp70 collaboration (Fig. 3, *A–C*). We tested whether these Hsp104 variants could confer induced thermotolerance *in vivo*. In preconditioned Δ*hsp104* cells, Hsp104^A430W^, Hsp104^L431W^, and Hsp104^D438W^ were severely defective in thermotolerance (Fig. 3, *D* and *E*). Indeed, Hsp104^A430W^ and Hsp104^L431W^ were completely inactive and conferred thermotolerance levels comparable with the vector control (Fig. 3, *D* and *E*). By contrast, Hsp104^D438W^ afforded some thermotolerance, but was greatly impaired compared with WT Hsp104 (Fig. 3*E*). Importantly, tryptophan substitutions in the distal loop or elsewhere in the MD did not impair thermotolerance: Hsp104^A437W^, Hsp104^A453W^, Hsp104^Y466W^, Hsp104^Y497W^, and Hsp104^Y507W^ (Fig. 1*C*) all conferred thermotolerance comparable with WT Hsp104 (Fig. 3*D*) (63). Thus, the inability of Hsp104^A430W^, Hsp104^L431W^, and Hsp104^D438W^ to confer thermotolerance was a specific effect of tryptophan substitution at precise positions in the distal loop. These Hsp104 variants were expressed at similar levels (data not shown). Thus, conserved residues in the MD distal loop of Hsp104 enable collaboration with Hsp70 and induced thermotolerance *in vivo*.

##### ClpB^A428W^ Is Unable to Collaborate with DnaK

To test conservation in ClpB, we generated ClpB^A428W^, which is analogous to Hsp104^A430W^ (Fig. 1*D*). The intrinsic disaggregase activity of ClpB^A428W^ was similar to WT ClpB. Indeed, ClpB^A428W^ was slightly more active than WT ClpB in reactivating aggregated luciferase in the presence of ATPγS and ATP (Fig. 3*F*) and inactive in the presence of ATP (Fig. 3*G*). However, ClpB^A428W^ was unable to collaborate with DnaK, DnaJ, and GrpE (Fig. 3*H*). Thus, the A428W mutation specifically ablates ClpB-DnaK collaboration. These data suggest that the function of the MD distal loop in Hsp70 collaboration is conserved across two billion years of evolution.

##### Why Are Specific Hsp104 Distal Loop Variants Selectively Impaired in Hsp70 Collaboration?

The inability of specific Hsp104 distal loop variants, *e.g.* Hsp104^A430W^, Hsp104^L431W^, and Hsp104^D438W^, to collaborate with Hsp70 could have at least three causes: (*a*) these distal loop variants may have an impaired ATPase cycle that precludes collaboration with Hsp70. Indeed, some ATPase-defective ClpB variants are less able to synergize with DnaK (41); (*b*) by analogy with the ClpC distal loop, which directly engages MecA (62), the distal loop of Hsp104 might directly interact with Hsp70 and facilitate collaboration. Mutations in this region could disrupt an Hsp70-MD interface and impair collaboration; and (*c*) mutations in the distal loop might alter an intramolecular Hsp104 interface that regulates Hsp70 collaboration. In fact, one hexameric model of Hsp104 and ClpB posits that the distal loop lies in close proximity to NBD2 (2, 50–52). Thus, mutations in the distal loop may interfere with NBD2-MD contacts in a way that selectively precludes Hsp70 collaboration.

##### Reduced ATPase Activity Does Not Readily Explain Inability to Collaborate with Hsp70

**FIGURE 4.**
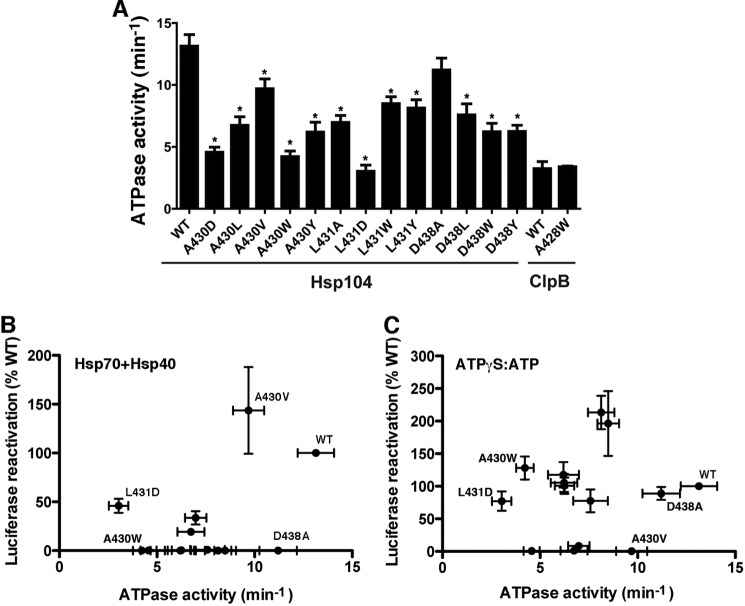
**Reduced ATPase activity does not explain the inability of specific distal loop variants to collaborate with Hsp70.** *A,* the indicated Hsp104 or ClpB variants (0.25 μm) were incubated for 12 (Hsp104 variants) or 30 min (ClpB variants) with ATP (1 mm) and the resulting ATPase rates were determined. Values represent mean ± S.E. (*n* = 3–9). A one-way analysis of variance with the post hoc Dunnett's multiple comparisons test was used to compare Hsp104^WT^ to Hsp104 variants. A *t* test was used to compare ClpB to ClpB^A428W^ (* denotes *p* < 0.05). *B,* reactivation of luciferase in the presence of Hsc70, and Hdj2 is plotted against ATPase activity for WT Hsp104 and variants. Positions of WT, A430W, A430V, L431D, and D438A are indicated. Values represent mean ± S.E. (*n* = 3–9). A simple linear regression yielded a coefficient of determination, *R*^2^ = 0.1994. *C,* reactivation of luciferase in the presence of ATPγS:ATP is plotted against ATPase activity for WT Hsp104 and variants. Positions of WT, A430W, A430V, L431D, and D438A are indicated. Values represent mean ± S.E. (*n* = 3–10). A simple linear regression yielded a coefficient of determination, *R*^2^ = 0.012.

We determined the ATPase activity of each Hsp104 distal loop mutant, as well as WT ClpB and ClpB^A428W^ (Fig. 4*A*). The majority of Hsp104 distal loop mutants had mildly impaired ATPase activity, ranging from ∼30% (Hsp104^L431D^: ∼3.04 ± 0.5 min^−1^) to ∼85% (Hsp104^D438A^: ∼11.2 ± 0.96 min^−1^) of WT Hsp104 activity (∼13.1 ± 0.93 min^−1^). In fact, impairment was statistically significant with the exception of Hsp104^D438A^ (Fig. 4*A*). Thus, at first glance, impaired ATPase activity of these mutants might explain defective collaboration with Hsp70 (Fig. 4*A*). However, upon closer inspection there is no simple linear correlation between ATPase activity and the ability to collaborate with Hsp70 (Fig. 4*B*, linear regression *R*^2^ = 0.1994). For example, Hsp104^D438A^ has ∼85% WT Hsp104 ATPase activity, but is unable to collaborate with Hsp70 (Fig. 4*B*), whereas Hsp104^L431D^ has the lowest ATPase activity but can collaborate with Hsp70 in luciferase reactivation (Figs. 3*C* and 4*B*). Moreover, ClpB^A428W^, which is unable to collaborate with DnaK (Fig. 3*H*), did not have lower ATPase activity than WT ClpB (compare ∼3.2 ± 0.57 min^−1^ for WT ClpB to ∼3.4 ± 0.06 min^−1^ for ClpB^A428W^; Fig. 4*A*). In fact, if simply decreasing ATPase activity led to defective Hsp70 collaboration, we would expect all distal loop variants to be deficient in Hsp70 collaboration. However, this is not the case. For example, Hsp104^A430V^ can collaborate effectively with Hsp70 (Figs. 3*C* and 4*B*). Thus, the moderately impaired ATPase activity of these distal loop variants does not explain their inability to collaborate with Hsp70.

There was also no simple linear relationship between the Hsp104 variant ATPase activity and ability to reactivate luciferase in the presence of ATPγS and ATP (Fig. 4*C*, linear regression, *R*^2^ = 0.012). Thus, the ATPase activity of these Hsp104 variants provides little information about their disaggregase activity in the presence of Hsp70 or ATPγS and ATP.

##### Isolated MD Does Not Interact with Hsp70 or Inhibit Disaggregation in Trans

Another possibility for how specific distal loop mutations impair Hsp70 collaboration is that mutation in this region disrupts a direct interaction between this region of Hsp104 and Hsp70. Indeed, in peptide arrays Hsp70 interacts with a distal loop peptide (residues 435–448) in this region (46). However, a direct interaction between this region of Hsp104 and Hsp70 could not be observed using a cross-linking strategy (46). To test for a direct interaction, we purified isolated Hsp104 MD as a N terminally GST-tagged protein. We immobilized the Hsp104 MD on glutathione-Sepharose and probed for a direct interaction with Hsp70 by affinity chromatography. We were unable to detect an interaction between the Hsp104 MD and Hsp70 (data not shown). Thus, the isolated Hsp104 MD does not appear to form a stable interaction with Hsp70. We may have failed to detect the putative interaction between the Hsp104 MD and Hsp70 because the interaction is too weak or too transient. Thus, we tested whether isolated Hsp104 MD might competitively inhibit the collaboration between Hsp70 and Hsp104 in *trans*. However, even a 100-fold molar excess of isolated Hsp104 MD had no effect on Hsp70-dependent disaggregation of luciferase by Hsp104 (data not shown). These experiments do not exclude the possibility that the MD of Hsp104 interacts directly with Hsp70. However, the isolated MD appears insufficient to recapitulate the Hsp104 and Hsp70 interaction.

##### Tryptophan Fluorescence Reveals That the Hsp104 Distal Loop Is Partially Buried

Next, we considered the third possibility for why distal loop mutation impairs Hsp70 collaboration. Mutations in the distal loop might alter an intramolecular Hsp104 interface that regulates Hsp70 collaboration. There is no atomic resolution structure of Hsp104, but the monomeric structure of ClpB from *T. thermophilus* has been solved (14). Yet, how monomers pack to form a hexamer is controversial and two conflicting models of the Hsp104 hexamer structure have been advanced based on cryo-EM reconstructions (2, 14, 49–53). In one of these, the MD projects out into solution away from the hexamer (2, 14, 49, 53). In this model, the distal loop appears poorly positioned to make intramolecular contacts with other domains of Hsp104 (2, 14, 49, 53). However, in the second model, the MD contacts both NBDs and the distal loop could make an intramolecular contact with NBD2 (2, 50–52).

**FIGURE 5.**
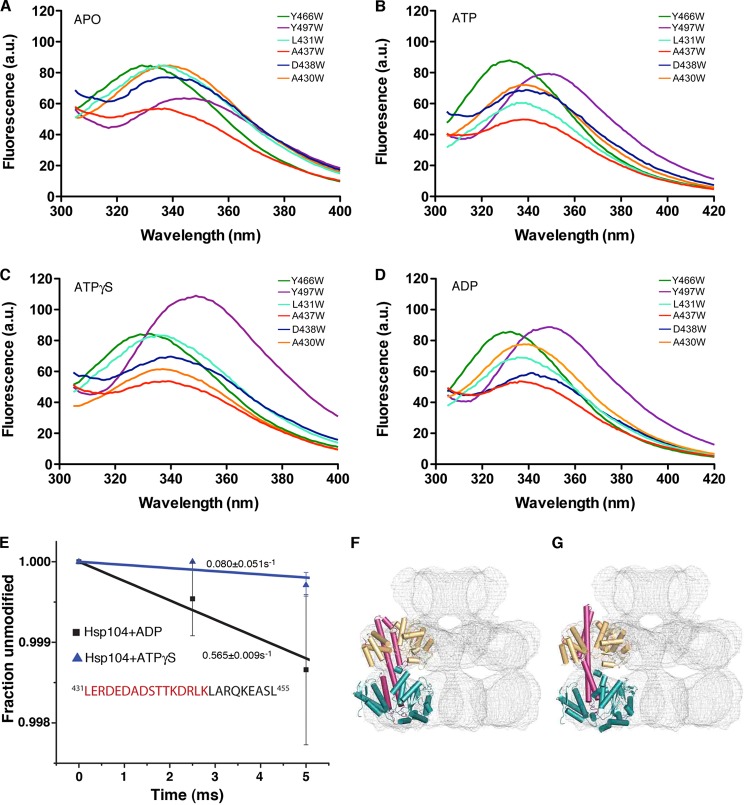
**The distal loop of the Hsp104 MD is partially solvent inaccessible.** *A–D*, tryptophan fluorescence spectra of Hsp104^Y466W^ (*green line*), Hsp104^Y497W^ (*purple line*), Hsp104^A430W^ (*orange line*), Hsp104^L431W^ (*light blue line*), Hsp104^A437W^ (*red line*), or Hsp104^D438W^ (*dark blue line*). All variants were left in the apo state (*A*) or incubated with ATP (*B*), ATPγS (*C*), or ADP (*D*) for 15 min prior to measurements. Samples were excited at 295 nm (4 nm bandwidth) and emission was collected from 305 to 505 nm (3 nm bandwidth). Each spectrum is an average of two readings. *E,* dose-response curves for Hsp104 peptide-(431–455) showing the fraction unmodified as a function of x-ray exposure time in the presence of ATPγS (*blue triangles*) or ADP (*black squares*). The distal loop residues are highlighted in *red*. Values represent mean ± S.E. (*n* = 3–4). *F* and *G,* comparison of published (*F*) (52) and an optimized (*G*) rigid body domain fit of Hsp104 homology model into the cryo-EM map of Hsp104^N728A^ in the presence of ATP. Only one subunit is depicted. Domains are color coded as follows: NDB1, *orange*; MD, *purple*; NBD2, *teal*. The resolution of the map does not allow for an unambiguous fit of the MD domain relative to NBD1 and NBD2. Possible fits are distinguished by a rotation of the MD around helix 2, which swaps the NBD2 contacts at the distal end of the MD. Cross-linking experiments confirmed the optimized fit (*G*) to be more accurate (see Figs. 6 and 7).

To assess the positioning of the MD within the Hsp104 hexamer, we exploited the fact that Hsp104 has no natural tryptophans, which makes it well suited for site-specific tryptophan fluorescence spectroscopy. This sensitive technique measures the polarity of the microenvironment within 10 Å of a Trp residue (64, 65). Red-shifted emission maxima (∼350 nm) indicate a polar microenvironment, *e.g.* solvated regions, whereas blue-shifted emission maxima (∼330 nm) indicate a more hydrophobic environment, *e.g.* regions that are shielded from solvent (64, 65). We mutated a number of MD residues to tryptophan and determined the fluorescence spectra of each mutant. As controls, we included the Tyr^466^ position (Fig. 1*C*), which is buried in both hexameric models and the Tyr^497^ position (Fig. 1*C*), which is solvated in both models to ensure that we could accurately determine the solvation state (49, 51, 52). Importantly, Hsp104^Y466W^ and Hsp104^Y497W^ confer WT levels of thermotolerance *in vivo* (Fig. 3*D*). As expected, in each nucleotide state tested (apo, ADP, ATP, and ATPγS) Hsp104^Y466W^ had blue-shifted emission maxima of ∼330 nm indicating a buried region, whereas Hsp104^Y497W^ had red-shifted emission maxima of ∼350 nm, indicating a solvated region (Fig. 5, *A–D*).

Hsp104^A430W^, Hsp104^L431W^, and Hsp104^D438W^ exhibited emission maxima between ∼335 and 340 nm in each nucleotide state (Fig. 5, *A–D*) indicating that the distal loop is partially shielded from solvent. These findings were not due to studying defective Hsp104 variants because Hsp104^A437W^, which displays WT levels of thermotolerance (Fig. 3*D*), also exhibited emission maxima between ∼336 and 339 nm (Fig. 5, *A–D*). Although fluorescence intensity changed in response to nucleotide for all the distal loop variants, we observed only minor shifts in emission maxima in response to different nucleotides, with the exception of Hsp104^L431W^ where the emission maximum remained at ∼335 nm for all states (Fig. 5, *A–D*). For example, the emission maximum of Hsp104^A430W^ in the absence of nucleotide and in the presence of ADP was more red-shifted (∼339 and ∼338 nm, respectively; Fig. 5, *A* and *B*), whereas the spectra of ATP and ATPγS were more blue-shifted (∼337 nm; Fig. 5, *C* and *D*). Likewise, the emission maximum of Hsp104^D438W^ in the presence of ADP was more red-shifted (∼341 nm; Fig. 5*D*), whereas in the presence of ATP or ATPγS it was more blue-shifted (∼339 and 340 nm, respectively; Fig. 5, *B* and *C*). Although minor, these shifts indicate that Ala^430^ and Asp^438^ are more solvated in the apo and ADP states and more buried in the ATP and ATPγS states.

Tryptophan fluorescence spectra reflect the average of the ensemble of protein conformers. Thus, the intermediate spectra of the distal loop variants can be interpreted in two ways: 1) the distal loop is partially sequestered from solvent all of the time; and 2) the distal loop moves between two different conformations, one of which is solvated and one of which is buried. Hsp104 hexamers are strongly asymmetric with each protomer adopting a distinct conformation (52). Thus, the distal loop could be buried in some subunits but solvent exposed in others. Regardless, the tryptophan emission spectra of Hsp104^A430W^, Hsp104^L431W^, Hsp104^A437W^, and Hsp104^D438W^ indicate that the distal loop is unlikely to always be solvent exposed, which is inconsistent with a model where the MD projects out from the hexamer into solvent in all nucleotide states examined (14, 49, 53).

##### X-ray Footprinting Confirms That Distal Loop Is Partially Buried

We confirmed that the MD distal loop of Hsp104 was partially buried in WT Hsp104 using synchrotron x-ray mediated hydroxyl radical footprinting, or x-ray footprinting (XF). In XF, millisecond bursts of x-rays generate hydroxyl radicals from solvent molecules (*i.e.* water). These radicals oxidatively modify solvent-exposed protein side chains in well defined ways, although Ala, Asp, and Asn have lower reactivity and Gly behaves like the main chain (66–68). For very short exposures, solvent-inaccessible regions of the protein are protected from modification (68). Methioninamide HCl is used to stop oxidative modification after exposure (66). Protease digestion of the irradiated sample followed by liquid chromatography electrospray ionization tandem mass spectrometry (MS/MS) allows identification of the modified, and thus solvent accessible regions of the protein. Certain nucleotides and buffers can scavenge free radicals and effectively quench oxidative damage. Moreover, high protein concentration or overexposure to x-rays can cause oxidation of protein that does not reflect solvent accessibility (66, 68). To circumvent these issues, Alexa 488, a dye that decreases in fluorescence upon oxidation by radiolysis, was used to calibrate ideal x-ray dosing conditions for specific Hsp104-nucleotide conditions (67). XF has been successfully used to test structural models of ClpA, a related hexameric AAA^+^ protein (69).

Having identified ideal x-ray dosing conditions, we exposed Hsp104 to white x-rays for 0–20 ms, in the presence of ADP or ATPγS. Samples were quenched, digested with pepsin, and processed for LC-ESI-MS/MS. MS/MS-verifiable coverage of pepsin-digested Hsp104 encompassed ∼85–90% of the protein. The modification rate of identified peptides was calculated by fitting a first order exponential decay curve extrapolated to zero. The complete XF dataset will be presented elsewhere.^7^ Here, we tracked a distal loop peptide (residues 431–455) and peptides spanning residues 456–476 that harbor Tyr^466^ in helix 2 (Fig. 1*C*). In agreement with the Hsp104^Y466W^ fluorescence data (Fig. 5*C*), peptide-(456–476) was not modified in the presence of ATPγS suggesting that this region is solvent inaccessible. In the presence of ADP, peptide-(456–466) was modified at time points longer than 2.5 ms, whereas peptide-(467–474) was unmodified. Although Tyr^466^ is predicted to be buried by both models (14, 49, 51, 52), the finding that this entire region (residues 456–476) is protected from modification in ATPγS is inconsistent with a model of Hsp104 hexamers where the MD projects out from the hexamer into solution (14, 49, 53).

In the presence of ADP and ATPγS, the distal loop peptide (residues 431–455) becomes oxidized indicating that it must be partially exposed to solvent (Fig. 5*E*). The rate of modification is ∼7-fold less rapid in ATPγS (∼0.080 s^−1^; Fig. 5*E*) than in ADP (∼0.565 s^−1^; Fig. 5*E*). Thus, the MD distal loop of WT Hsp104 is less solvent accessible in the presence of ATPγS and more solvent accessible in the presence of ADP, which is consistent with Hsp104^A430W^ and Hsp104^D438W^ fluorescence (Fig. 5, *C* and *D*). The MD distal loop of Hsp104 likely shifts from a more buried position to a more solvent exposed position upon ATP hydrolysis. These XF and tryptophan fluorescence data are consistent with a hexameric model in which the MD distal loop is positioned in a way that could make an intramolecular contact with NBD2 (2, 50–52).

##### Engineered Disulfides Establish That Helix 2 and the Distal Loop of the MD Contact NBD2

Using tryptophan fluorescence and XF, we have established that the distal loop of Hsp104 can become shielded from solvent. Size exclusion chromatography coupled to dynamic light scattering reveal that Hsp104 hexamers are monodisperse in solution (data not shown). Thus, the distal loop of the MD is likely to contact another domain within the Hsp104 hexamer. Where does the distal loop become buried?

To answer this question, we examined an optimized structural model of the Hsp104^N728A^ hexamer in the presence of ATP, where the distal loop is in close proximity to NBD2 (Fig. 5, *F* and *G*) (50–52). Based upon this optimized model (Fig. 5*G*), we site specifically mutated proposed contact residues between MD and NBD2 to cysteine and assessed the proximity by attempting to form disulfide cross-links. This strategy has confirmed that helix 3 of the MD can contact NBD1 in ClpB (14, 47). First, however, to avoid confounding effects of the six naturally occurring cysteines in Hsp104, we generated an Hsp104 variant, Hsp104^6CS^, where all six cysteines are mutated to serine. Hsp104^6CS^ retained approximately WT levels of the induced thermotolerance *in vivo* and luciferase disaggregation *in vitro* (data not shown).

**FIGURE 6.**
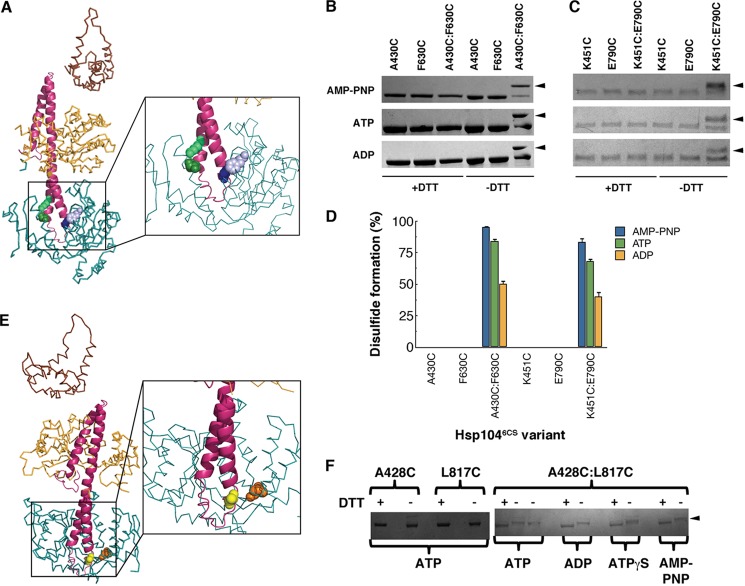
**The distal loop of the MD can contact NBD2 in Hsp104 and ClpB.** *A,* successful disulfide cross-links formed between the MD (*pink*) and NBD2 (*blue*) of Hsp104 (only one monomer shown) as predicted by the optimized Hsp104 rigid body fit of the Hsp104^N728A^ hexamer in the presence of ATP (Fig. 5*G*) (52). Residue A430C (*dark purple spheres*) in the MD could cross-link to F630C (*light purple spheres*) in NBD2. Residue K451C (*dark green spheres*) in the MD could cross-link to E790C (*light green spheres*) in NBD2. *B* and *C,* intramolecular disulfide cross-links formed between A430C and F630C (*B*) and K451C and E790C (*C*) in the presence of ADP, ATP, or AMP-PNP. Reduced and oxidized Hsp104 variants (±DTT) were analyzed by non-reducing SDS-PAGE. Cross-links were visualized by band up-shift in SDS-PAGE (*arrowheads* indicated up-shifted species). Single cysteine mutant controls did not show cross-linking. Up-shift was seen only in double cysteine mutants. *D,* the extent of disulfide formation monitored as in *B* and *C* was quantified using Ellman's reagent. Values represent mean ± S.E. (*n* = 3). *E,* successful disulfide cross-links formed between the MD (*pink*) and NBD2 (*blue*) of ClpB (only one monomer shown) as deduced from the optimized Hsp104 rigid body fit of the Hsp104^N728A^ hexamer in the presence of ATP (52). Residue A428C (*yellow spheres*) in the MD could cross-link to L817C (*orange spheres*) in NBD2. *F,* intramolecular disulfide cross-links formed between A428C and L817C in the presence of ADP, ATP, ATPγS, or AMP-PNP. Reduced and oxidized ClpB variants (±DTT) were analyzed by non-reducing SDS-PAGE. Cross-links were visualized by band up-shift in SDS-PAGE. Single cysteine mutant controls did not show cross-linking. Up-shift was seen only in double cysteine mutants (*arrowhead* indicates up-shifted species).

We introduced cysteine pairs into the Hsp104^6CS^ background to test the proposed proximity of A430C(MD)/F630C(NBD2) (Fig. 6*A, blue* and *mauve* residues) and K451C(MD)/E790C(NBD2) (Fig. 6*A*, *green* residues) (50–52). Disulfides were induced by diamide and detected by reduced mobility on non-reducing SDS-PAGE and with Ellman's reagent to quantify free sulfhydryls (47). Single cysteine variants served as negative controls. Disulfide cross-links readily formed between the MD residue A430C and NBD2 residue F630C (Fig. 6, *A* and *B*) as well as between MD residue K451C and the NBD2 residue E790C (Fig. 6, *A* and *C*). Both disulfides were intramolecular as no higher order multimeric species were observed in single or double cysteine variants. In both cases, the disulfide formed more readily in the presence of AMP-PNP (a non-hydrolyzable ATP analog) or ATP than in the presence of ADP (Fig. 6*D*). Thus, upon ATP binding the distal loop (Ala^430^) and helix 2 (Lys^451^) of the MD contact NBD2. Upon ATP hydrolysis, the MD is in less close contact with NBD2, which is in accord with tryptophan fluorescence (Fig. 5, *C* and *D*) and XF data (Fig. 5*E*). These findings argue against hexameric models where the MD invariably projects away from the hexamer out into solution (14, 49, 53) or where the distal loop invariably contacts NBD1 of an adjacent subunit (48), which would both leave the distal loop too distant from NBD2 to form disulfides.

##### The MD Distal Loop Contacts NBD2 in ClpB

Next, we determined if the NBD2-MD contacts were conserved in ClpB. Thus, we introduced pairs of cysteine residues into ClpB. However, we were unable to form a disulfide between A428C (A430C in Hsp104) and F621C (F630C in Hsp104) in ClpB. By contrast, in ClpB, A428C (Fig. 6*E*, *yellow* residue) readily formed a disulfide with L817C (Fig. 6*E*, *orange* reside), which is located in the NBD2 small domain (Fig. 6, *E* and *F*). Unlike Hsp104, we observed maximal disulfide formation in all nucleotide states (Fig. 6*F*). Thus, the MD is oriented differently in Hsp104 and ClpB. In comparison to Hsp104 the MD of ClpB seems to be rotated ∼90° anticlockwise around helix 2. Nonetheless, the MD distal loop also contacts NBD2 in ClpB, which further argues against a hexameric model where the MD invariably projects away from the hexamer out into solution (14, 49, 53) or where the distal loop invariably contacts NBD1 of an adjacent subunit (48).

##### NBD1-MD Contacts Are Not Conserved between ClpB and Hsp104

**FIGURE 7.**
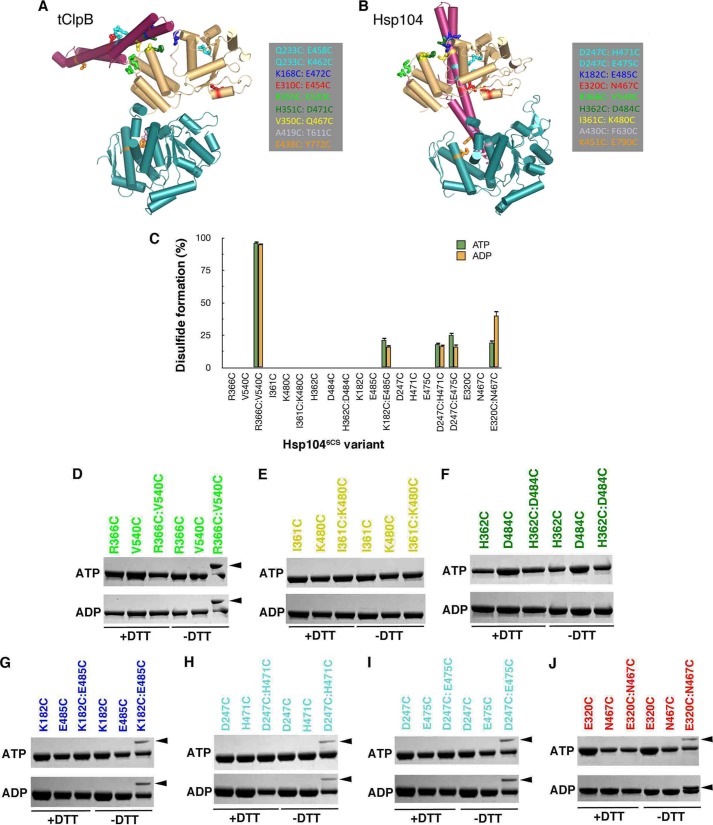
**Defining NBD1-MD contacts in Hsp104.** *A* and *B*, location of cross-linking pairs in the tClpB crystal structure (*A*) (14) compared with the homologous residues in the optimized Hsp104 rigid body fit of the Hsp104^N728A^ hexamer in the presence of ATP (*B*) (Fig. 5*G*) (52). Color-coded cross-linking pairs are listed in the *insets* and shown as *sticks* in the structures. The N-terminal domains are not shown for clarity. The MD is colored *purple*, NBD1 is colored *yellow*, and NBD2 is colored *blue. C,* disulfide cross-link formation of the indicated Hsp104^C6S^ variant was quantified using Ellman's reagent. Values represent mean ± S.E. (*n* = 2). *D–J*, reduced and oxidized Hsp104 variants (±DTT) were analyzed by non-reducing SDS-PAGE. Intramolecular disulfide cross-links were visualized by band up-shift in SDS-PAGE. Single cysteine mutant controls did not show cross-linking (*D–J*). Up-shift was seen only in specific double cysteine mutants: R336C/V540C (*D*), K182C/E485C (*G*), D247C/H471C (*H*), D247C/E475C (*I*), and E320C/N467C (*J*). Two double cysteine mutants did not show any up-shift: I361C/K480C (*E*) and H362C/D484C (*F*).

The NBD2-MD contacts in Hsp104 and ClpB are not anticipated by a hexameric model of Hsp104 based on the *T. thermophilus* ClpB (tClpB) crystal structure (Fig. 7*A*) (14, 49, 53), but are predicted by an Hsp104 cryo-EM model (Fig. 7*B*) (50–52). This discrepancy prompted us to test whether other engineered disulfides between the MD and NBD1 and within NBD1 of ClpB that were predicted and verified in tClpB could also be formed in Hsp104 (Fig. 7*A*) (14). Thus, we assessed the following cysteine pairs in Hsp104^6CS^, I361C(NBD1)/K480C(MD) and R366C(NBD1)/V540C(NBD1), which correspond to V350C(NBD1)/Q467C(MD) (Fig. 7, *A* and *B*, *yellow* residues) and R355C(NBD1)/E520C(NBD1) (Fig. 7, *A* and *B*, *light green* residues) in tClpB, respectively (14). I361C(NBD1)/K480C(MD) are predicted to form a disulfide in the tClpB model (Fig. 7*A*, *yellow* residues), but not the other model (Fig. 7*B*, *yellow* residues), whereas R366C(NBD1)/V540C(NBD1) are predicted to form a disulfide in both (Fig. 7, *A* and *B*, *light green* residues). In agreement with data from tClpB (14), R366C(NBD1)/V540C(NBD1) in Hsp104^6CS^ readily formed a disulfide within NBD1 (Fig. 7, *C* and *D*). Thus, two β-strands of the NBD1 small domain, which are separated in primary sequence by the entire MD, can be linked by a disulfide in Hsp104 and ClpB (14). The cross-link formed extremely effectively in ADP or ATP (Fig. 7, *C* and *D*). By contrast and as predicted by the Hsp104 cryo-EM model (Fig. 7*B*) (50–52), disulfides did not form between I361C(NBD1)/K480C(MD) in ATP or ADP (Fig. 7, *C* and *E*). Thus, NBD1-MD domain contacts are not absolutely conserved between ClpB and Hsp104.

##### Defining NBD1-MD Contacts in Hsp104

To define NBD1-MD contacts in Hsp104 we tested predictions from an optimized cryo-EM hexameric model of Hsp104 (Fig. 5*G*) (50–52). We assessed disulfide formation between K182C(NBD1)/E485C(MD) (Fig. 7*B*, *dark blue* residues), D247C(NBD1)/H471C(MD) (Fig. 7*B*, *cyan* residues), D247C(NBD1)/E475C(MD) (*cyan* residues Fig. 7*B*), and E320C(NBD1)/N467C(MD) (Fig. 7*B*, *red* residues) in Hsp104^6CS^. We also assessed H362C(NBD1)/D484C(MD) (Fig. 7*B*, *dark green* residues), which are not predicted to be in close enough proximity to form disulfides (50–52). Indeed, H362C(NBD1)/D484C(MD) did not form disulfides in ATP or ADP (Fig. 7, *C* and *F*). In the presence of ADP or ATP, K182C(NBD1)/E485C(MD), D247C(NBD1)/H471C(MD), and D247C(NBD1)/E475C(MD) all formed intramolecular disulfides, although the efficiency was ∼15–20%, indicating that on average only one subunit per hexamer could make this cross-link (Fig. 7, *C*, *G*, *H*, and *I*). By contrast, the E320C(NBD1)/N467C(MD) disulfide formed with greater efficiency in ADP (∼40%) than ATP (∼15%) (Fig. 7, *C* and *J*). None of these disulfides are expected by a hexameric model where the MD projects out into solution (Fig. 7*A*) (14, 49, 53). Thus, upon ATP hydrolysis the MD shifts position such that Ala^430^ in the distal loop moves away from Phe^630^ in NBD2 and simultaneously Asn^467^ in helix 2 moves closer to Glu^320^ of NBD1.

##### Movement of the MD Distal Loop Away from NBD2 Is Critical for Disaggregation

**FIGURE 8.**
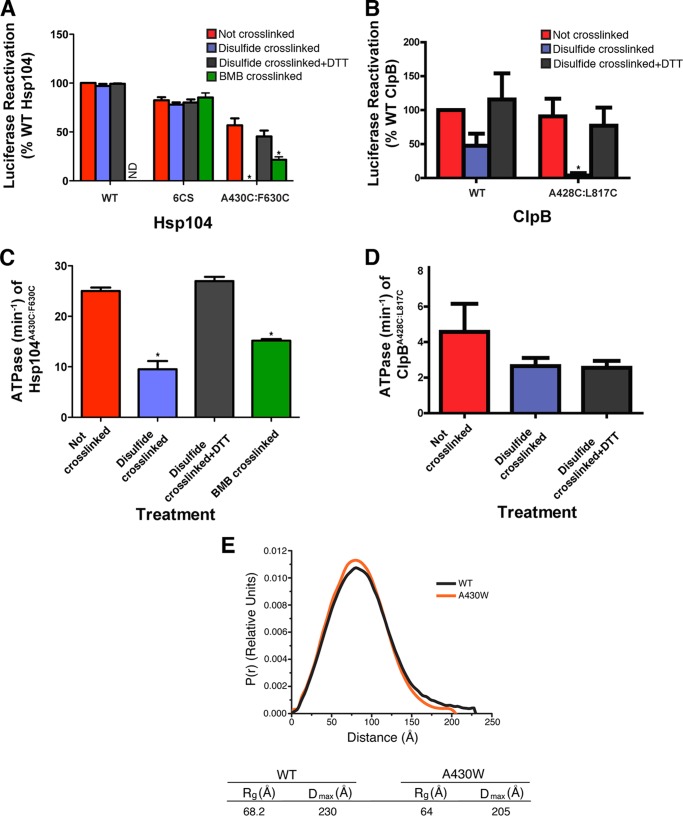
**Cross-linking NBD2 to the distal loop diminishes disaggregase activity of Hsp104 and ClpB.** *A,* urea-denatured firefly luciferase aggregates were incubated for 90 min at 25 °C with Hsp104 (1 μm), Hsp104^C6S^ (1 μm), or Hsp104^6CS/A430C/F630C^ in the presence of ATP (5.1 mm), Hsc70 (1 μm), and Hdj2 (1 μm). Hsp104 variants were either not cross-linked (*red*), disulfide cross-linked with no DTT (*blue*), disulfide cross-linked and subsequently reduced with DTT (*gray*), or BMB cross-linked (*green*). The effect of BMB cross-linking on WT Hsp104 was not determined (denoted by *ND*). Reactivation of luciferase was then determined by luminescence and converted to % WT disassembly activity (activity of 1 μm WT Hsp104 in the presence of Hsc70 and Hdj2). Values represent mean ± S.E. (*n* = 3). A one-way analysis of variance was performed to compare the means from each condition for each Hsp104 protein. The post hoc Dunnett's multiple comparisons test was used to compare the not cross-linked control to the disulfide-linked, disulfide-linked + DTT, and BMB cross-linked conditions. * denotes *p* < 0.05. *B,* urea-denatured firefly luciferase aggregates were incubated for 90 min at 25 °C with ClpB (0.167 μm), or ClpB^A428C/L817C^ (0.167 μm) in the presence of ATP (5.1 mm), DnaK (1 μm), DnaJ (0.033 μm), and GrpE (0.0167 μm). ClpB variants were not cross-linked (*red*), disulfide cross-linked with no DTT (*blue*), or disulfide cross-linked and subsequently reduced with DTT (*gray*). Reactivation of luciferase was then determined by luminescence and converted to % WT disassembly activity (activity of 1 μm WT ClpB in the presence of DnaK, DnaJ, and GrpE). Values represent mean ± S.E. (*n* = 3). A one-way ANOVA was performed to compare the mean from each condition for each ClpB protein. The post hoc Dunnett's multiple comparisons test was used to compare the not cross-linked control to the disulfide-linked, disulfide-linked + DTT, and BMB cross-linked conditions. * denotes *p* < 0.05. *C*, ATPase activity of Hsp104^6CS/A430C/F630C^ after no cross-linking (*red*), disulfide cross-linking (*blue*), disulfide cross-linking followed by reduction with DTT (*gray*), or BMB cross-linking (*green*). Values represent mean ± S.E. (*n* = 3). A one-way analysis of variance was performed to compare the means from each condition. The post hoc Dunnett's multiple comparisons test was used to compare the not cross-linked control to the disulfide-linked, disulfide-linked + DTT, and BMB cross-linked conditions. * denotes *p* < 0.05. *D,* ATPase activity of ClpB^A428C/L817C^ after no cross-linking (*red*), disulfide cross-linking (*blue*), or disulfide cross-linking followed by reduction with DTT (*gray*). Values represent mean ± S.E. (*n* = 3). A one-way analysis of variance was performed to compare the means from each condition. The post hoc Dunnett's multiple comparisons test was used to compare the not cross-linked control to the disulfide-linked, and disulfide-linked + DTT. No significant differences were found. *E,* the A430W mutation in the distal loop changes the shape and compacts the Hsp104 hexamer. The *P*(*r*) distribution for WT Hsp104 (black line) and Hsp104^A430W^ (*orange line*) in the presence of ATP derived from SAXS data. The *P*(*r*) curves have been normalized to the area under the curve. The *D*_max_ and *R_g_* values are summarized below (see “Experimental Procedures” and Table 1).

The cross-linking data suggested that the MD populates two different conformations that result in the distal loop being in two different environments. The first conformation (populated in the ATP-bound state) results in a more solvent-inaccessible distal loop that is juxta-NBD2 and thus able to form a covalent cross-link between the two domains. The second conformation (populated in the ADP-bound state) leaves the distal loop more solvent accessible and not as closely associated with NBD2. To probe the functional importance of the NBD2-MD contact we assessed the luciferase disaggregase and ATPase activity of disulfide cross-linked and uncross-linked Hsp104^6CS/A430C/F630C^ and ClpB^A428C/L817C^. We also cross-linked A430C to F630C in the Hsp104^6CS^ background with the longer and more flexible 11-Å linker, BMB. Before cross-linking, Hsp104^6CS/A430C/F630C^ and ClpB^A428C/L817C^ displayed luciferase reactivation levels comparable with WT (Fig. 8, *A* and *B*, *red bars*). After disulfide cross-linking, Hsp104^6CS/A430C/F630C^ and ClpB^A428C/L817C^ had very low luciferase reactivation activity, suggesting that the cross-links nixed disaggregase activity (Fig. 8, *A* and *B*, *blue bars*). Importantly, reduction of the Hsp104^6CS/A430C/F630C^ and ClpB^A428C/L817C^ disulfides restored disaggregase activity (Fig. 8, *A* and *B*, *gray bars*). Thus, the cross-linking procedure itself does not result in impaired activity. We confirmed by SDS-PAGE that BMB induced an upshift indicative of an intramolecular cross-link in ∼85% of total Hsp104^6CS/A430C/F630C^. In contrast to disulfide cross-linked Hsp104^6CS/A430C/F630C^, BMB cross-linked Hsp104^6CS/A430C/F630C^ retained some disaggregase activity, whereas Hsp104^6CS^ was unaffected (Fig. 8*A*, *green bars*). Thus, a longer, more flexible cross-link between NBD2 and the distal loop permits some disaggregase activity.

To determine how the disulfide cross-link between MD and NBD2 inhibits disaggregase activity, we examined the ATPase activity of Hsp104^6CS/A430C/F630C^ and ClpB^A428C/L817C^. Hsp104^6CS/A430C/F630C^ displayed an elevated ATPase rate (∼25 ± 0.67 min^−1^) compared with WT Hsp104 (∼13.1 ± 0.93 min^−1^) (Fig. 8*C*, *red bar*). Disulfide cross-linked Hsp104^6CS/A430C/F630C^ had reduced ATPase activity (∼9.54 ± 1.63 min^−1^) (Fig. 8*C*, *blue bar*). Inhibition was relieved upon reduction of the Hsp104^6CS/A430C/F630C^ disulfide (∼27 ± 0.67 min^−1^), indicating that the NBD2-MD disulfide contact inhibits ATP hydrolysis (Fig. 8*C*, *gray bar*). BMB cross-linked Hsp104^6CS/A430C/F630C^ was less inhibited (∼15.2 ± 0.3 min^−1^) (Fig. 8*C*, *green bar*). By contrast, disulfide cross-linked ClpB^A428C/L817C^ had similar ATPase activity to uncross-linked ClpB^A428C/L817C^ (Fig. 8*D*). Thus, in ClpB, constraining the distal loop contact with NBD2 inhibits disaggregase activity (Fig. 8*B*) without significantly affecting ATPase activity. Collectively, the foregoing data suggest that the distal loop of the MD and NBD2 of the same subunit are in close proximity and can form an autoinhibitory contact that tightly constrains the disaggregase activity of Hsp104 and ClpB. Based on these observations, we suggest that a critical step in the activation of Hsp104 and ClpB for protein disaggregation lies in the separation of these autoinhibitory NBD2-MD contacts.

##### The A430W Mutation Changes the Shape and Compacts the Hsp104 Hexamer

To determine how the distal loop mutants might affect Hsp104 hexamer structure, we performed SAXS and WAXS to determine the maximum dimension (*D*_max_) and radius of gyration (*R_g_*) of WT Hsp104 and Hsp104^A430W^ hexamers in the presence of ATP (Table 1; Fig. 8*E*). SAXS exploits the fact that x-ray scattering by a particle at very low angles contains information about particle size and shape. The pairwise distance distribution (*P*(*r*) curve), which represents the distances between pairs of atoms within a given volume, reveals that Hsp104^A430W^ has a smaller *D*_max_ than WT Hsp104 (205 Å compared with 230 Å for WT Hsp104). Moreover, the *R_g_*, which is the root mean square average of all of the interatomic distances with respect to the center-of-mass, is shorter for Hsp104^A430W^ than for WT Hsp104 (compare 64 to 68.2 Å) (Table 1, Fig. 8*E*). Thus, Hsp104^A430W^ occupies a smaller spatial extent in solution than WT Hsp104 (Table 1). The smaller spatial extent of Hsp104^A430W^ is evident in the *P*(*r*) curves where WT Hsp104 exhibits a decrease in smaller interatomic vectors near the *R_g_* (the peak) and an increase in larger interatomic vectors (Fig. 8*E*). The Porod volume of Hsp104^A430W^ is also smaller than that of WT Hsp104 (Table 1). Porod-Debye analysis (70) indicated that large changes in flexibility do not underlie these differences (Porod exponents, P*_x_*, of 3.9 and 3.7 for the WT Hsp104 and Hsp104^A430W^ hexamers, respectively; Table 1). Thus, the A430W mutation likely stabilizes the NBD2-MD contact in a way that yields a more compact hexamer, which occupies a smaller spatial extent and volume and cannot collaborate with Hsp70.

**TABLE 1:** **Parameters derived from SAXS for WT Hsp104 and Hsp104^A430W^ in the presence of ATP** An *R_g_* from the Guinier region of the scattering curve was determined using the program PRIMUS (59). Distance distribution functions *P*(*r*) were calculated by the program GNOM using an indirect Fourier transform (61). The maximum dimension of the particle (*D*_max_) was determined by examining the quality of fit to the experimental data for a *D*_max_ range of 180 to 280 Å, varied in 5-Å increments. Values for *R_g_* were computed from the second moment of the *P*(*r*). The Porod volume and *p* value were calculated by the java-based program ScÅtter (www.bioisis.net/tutorial/9). The mass of the particle was calculated from Q*_r_* as described (71). See also “Experimental Procedures.”

Location	Concentration	Guinier				GNOM				Porod		MM by Q*_r_*	
		*q*_min_	q*R_g_* range	*R_g_*	I(O)	Angle range	*D*_max_	*R_g_*	I(O)	Volume	P	Exp MM	Theor MM
**WT ATP**													
NSLS	2.5 mg/ml	0.01	0.68–1.49	67.7 ± 0.07	450.12 ±0.46	0.01–0.83	220	67.1	445	1,654,371	3.9	631,000	612,000
NSLS	5.0 mg/ml	0.01	0.75–1.50	68.1 ±0.05	936.03 ± 0.69	0.01–0.81	230	68.2	935	1,674,270	3.9	645,000	612,000

**A430W ATP**													
NSLS	1.5 mg/ml	0.01	0.77–1.53	63.8 ± 0.09	173.85 ± 0.26	0.01–0.30	200	63.1	173	1,569,217	3.6	596,000	612,000
NSLS	2.1 mg/ml	0.01	0.59–1.50	65.1 ± 0.07	403.11 ± 0.45	0.01–0.79	205	64	398	1,595,613	3.7	589,000	612,000

##### Hsp104 Has a Less Stringent Requirement for Hsp70 Collaboration Than ClpB

**FIGURE 9.**
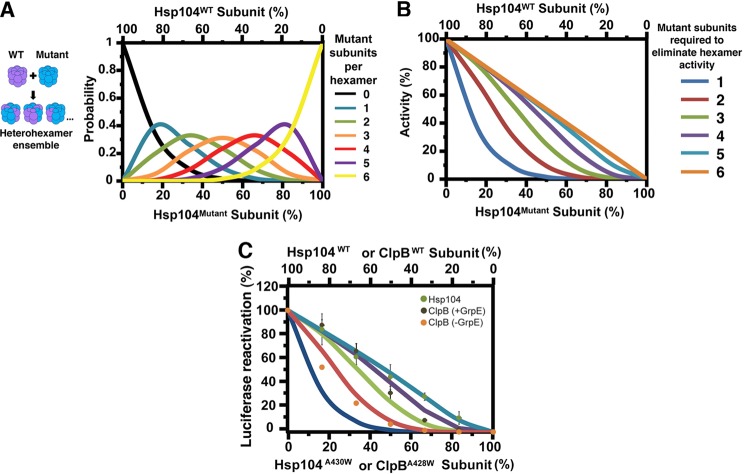
**For disordered aggregate dissolution, Hsp104 hexamers require fewer subunits able to collaborate with Hsp70 than ClpB.** *A*, theoretical ensembles of Hsp104 hexamers containing no mutant (*black*), one mutant subunit (*blue*), two (*green*), three (*orange*), four (*red*), five (*purple*), and six mutant subunits (*yellow*) as a function of the percentage of mutant subunits present. See “Experimental Procedures” for more details. *B,* theoretical activity curves for mutant doping experiments. The activity varies as a function of the percentage of mutant subunits present assuming that hexamer activity is proportional to the number of active subunits and all subunits in a hexamer are inactive when a specified number of mutant subunits per hexamer is breached. Curves are shown for situations where one or more mutant subunits (*blue*), two or more mutant subunits (*red*), three or more mutant subunits (*green*), four or more mutant subunits (*purple*), five or more mutant subunits (*light blue*), or six mutant subunits (*orange*) are required to eliminate hexamer activity. See “Experimental Procedures” for more details. *C,* urea-denatured luciferase aggregates were treated with Hsp104, Hsc70, and Hdj2 plus increasing fractions of Hsp104^A430W^ (*green markers*). Alternatively, luciferase aggregates were treated with ClpB, ClpB^A428W^, DnaK, and DnaJ. GrpE was included (*gray markers*) or omitted (*orange markers*). Where error bars are not visible, they are too small and are hidden by the marker. Luciferase reactivation (% WT activity) was then assessed. Values represent mean ± S.E. (*n* = 3–4). *Solid lines* represent theoretical activity curves for situations where one or more mutant subunits (*blue*), two or more mutant subunits (*red*), three or more mutant subunits (*green*), four or more mutant subunits (*purple*), or five or more mutant subunits (*light blue*) are required to eliminate hexamer activity.

Hsp104 and ClpB form dynamic hexamers, which exchange subunits rapidly and randomly. Thus, we can use a mutant-doping strategy to ask how many subunits per hexamer are required for a given aspect of Hsp104 or ClpB activity (26, 38). Here, WT subunits are mixed with subunits that contain a mutation that specifically abolishes one aspect of activity (*e.g.* Hsp70 collaboration). These mixtures rapidly form heterohexamers containing both WT and mutant subunits according to the binomial distribution (Fig. 9*A*) (26, 38). By applying these heterohexamer ensembles to reactivate disordered luciferase aggregates, we obtain a measure of how many WT subunits per hexamer are required for a specific activity (*e.g.* Hsp70 collaboration) (Fig. 9*B*). For example, by mixing WT Hsp104 with Hsp104^A430W^ or WT ClpB with ClpB^A428W^ in known and established ratios we were able to determine how many WT subunits per hexamer are required for Hsp104 or ClpB to collaborate with Hsp70 during disaggregation. We conducted doping experiments (see “Experimental Procedures”) with Hsp104^A430W^ and ClpBA^428W^. ClpB was more sensitive to the Hsp70 collaboration-deficient mutant than Hsp104. In fact, ClpB required between 3 and 4 WT subunits per hexamer to disaggregate luciferase (Fig. 9*C*, *gray* markers), whereas Hsp104 only required 2 WT subunits per hexamer (Fig. 9*C*, *green* markers).

We were concerned that the differences observed between Hsp104 and ClpB might be due to GrpE, a nucleotide exchange factor for DnaK, which is included in ClpB-mediated reactivation, but not in the Hsp104 experiments. To determine whether GrpE could account for the observed differences, we repeated the ClpB^A428W^ doping experiments, but omitted GrpE. Here, we observed an even more deleterious effect upon titration of ClpB^A428W^. In the absence of GrpE, ClpB required between 5 and 6 WT subunits per hexamer to accomplish disaggregation (Fig. 9*C*, *orange* markers). Thus, more ClpB subunits per hexamer must be competent to collaborate with Hsp70 than Hsp104 subunits for successful disaggregation. Since these mutant doping studies of *in vitro* luciferase reactivation by Hsp104 and ClpB accurately model physiological conditions (26), we suggest that Hsp104 and ClpB hexamers differ fundamentally in how they collaborate with Hsp70.

## DISCUSSION

Here, we establish for the first time that conserved residues in the MD distal loop of Hsp104 and ClpB are specifically critical for collaboration with Hsp70. We establish that the MD distal loop forms an autoinhibitory intrasubunit contact with NBD2, which is consistent with some hexameric models of Hsp104 (50–52), but not others (14, 48, 49, 53). Recently, the MD of Hsp104 and ClpB has been implicated as being crucial for collaboration with Hsp70 (44–46, 48). To isolate Hsp104 and ClpB variants specifically defective in Hsp70 collaboration we exploited the fact that Hsp104 and ClpB resolve disordered aggregates in the absence of Hsp70 when supplemented with mixtures of ATPγS and ATP, and are inactive with ATP alone. Thus, we could exclude pleiotropic mutants, such as Hsp104^Y507A^, which were active with ATP alone in the absence of Hsp70 and Hsp40, and inactive in the presence of ATPγS and ATP. Introducing Trp or Tyr at the highly conserved 430 or 431 positions or introducing Ala, Leu, Trp, or Tyr at the 438 position in Hsp104, specifically ablated Hsp70 collaboration, without activating Hsp104 in the presence of ATP alone or perturbing disaggregase activity in the presence of ATPγS and ATP. This function of the MD distal loop in Hsp70 collaboration is conserved in ClpB.

Why are some distal loop mutants (*e.g.* Hsp104^A430W^, ClpB^A428W^) unable to collaborate with Hsp70? These mutants form hexamers, retain WT secondary structure, possess robust ATPase activity, and disaggregate disordered aggregates in the presence of permissive ATPγS:ATP ratios. The MD distal loop does not engage Hsp70 directly (44–46, 48). Rather, it makes intra-subunit contacts with NBD2, which is consistent with one hexameric model of Hsp104 (51, 52) but not with models where the MD invariably projects out into solution (14, 49, 53) or where the distal loop invariably contacts NBD1 of the neighboring subunit (48). When the NBDs are populated with ATP or ATPγS, the distal loop of the MD is less solvent accessible and when ADP is present, the distal loop is more solvent accessible. In Hsp104, upon ATP hydrolysis the MD shifts such that Ala^430^ in the distal loop moves away from Phe^630^ in NBD2 and at the same time Asn^467^ in helix 2 moves into closer proximity with Glu^320^ of NBD1. We suggest that the NBD2-MD interface is autoinhibitory and must separate for successful protein disaggregation. Constraining the interface with disulfide cross-links but not longer BMB cross-links prevents protein disaggregation in the presence of Hsp70 and Hsp40. Distal loop variants, such as Hsp104^A430W^, may mimic the disulfide cross-linking effect by stabilizing the distal loop and NBD2 interaction. In fact, Hsp104^A430W^ hexamers are more compact, which may be due to a tighter interaction between the distal loop and NBD2.

We used Hsp104^A430W^ and ClpB^A428W^ in subunit doping experiments (26) to determine how many Hsp70-collaboration competent subunits are required per hexamer to disaggregate disordered substrates. We determined that Hsp104 only requires 2 WT subunits to collaborate with Hsp70, whereas ClpB required between 3 and 4 WT subunits in the presence of GrpE and 5 to 6 subunits in the absence of GrpE (Fig. 9*C*). Thus, ClpB has a more rigid requirement for Hsp70 collaboration than Hsp104, and this requirement is intensified in the absence of GrpE. This result was surprising because it indicated that Hsp104 and ClpB differ fundamentally in their mechanism of collaboration with Hsp70. This fundamental difference between Hsp104 and ClpB, defined here with pure components, is likely to explain differences between Hsp104 and ClpB observed under physiological conditions. More ClpB subunits per hexamer must be competent to interact functionally with Hsp70 than Hsp104 subunits. This distinction likely reflects important differences in how substrate handover from Hsp70 to ClpB and Hsp104 is coordinated. For example, more ClpB subunits per hexamer are likely required to wrench substrate from Hsp70, particularly in the absence of GrpE. Moreover, ClpB is more dependent upon Hsp70 for optimal activity than Hsp104 (Fig. 2, *C* and *D*). These differences between Hsp104 and ClpB likely reflect differential tuning for the respective challenges of yeast and bacterial proteostasis.

## Footnotes

7 E. A. Sweeny and J. Shorter, manuscript in preparation.

## Footnotes

6 The abbreviations used are:

NBD: nucleotide-binding domain
MD: middle domain
BMB: 1,4-bis-maleimidobutane
WAXS: wide angle x-ray scattering
SAXS: small angle x-ray scattering
XF: x-ray footprinting
ATPγS: adenosine 5′-*O*-(thiotriphosphate)
AMP-PNP: adenosine 5′-(β,γ-imino)triphosphate.
